# Unraveling the Planar-Globular Transition in Gold Nanoclusters through Evolutionary Search

**DOI:** 10.1038/srep34974

**Published:** 2016-11-28

**Authors:** Alper Kinaci, Badri Narayanan, Fatih G. Sen, Michael J. Davis, Stephen K. Gray, Subramanian K. R. S. Sankaranarayanan, Maria K. Y. Chan

**Affiliations:** 1 Center for Nanoscale Materials, Argonne National Laboratory, Lemont, IL 60439, USA; 2 Chemical Sciences Division, Argonne National Laboratory, Lemont, IL 60439, USA

## Abstract

Au nanoclusters are of technological relevance for catalysis, photonics, sensors, and of fundamental scientific interest owing to planar to globular structural transformation at an anomalously high number of atoms i.e. in the range 12–14. The nature and causes of this transition remain a mystery. In order to unravel this conundrum, high throughput density functional theory (DFT) calculations, coupled with a global structural optimization scheme based on a modified genetic algorithm (GA) are conducted. More than 20,000 Au_12_, Au_13_, and Au_14_ nanoclusters are evaluated. With any DFT functional, globular and planar structures coexist across the size range of interest. The planar-globular transition is gradual at room temperature rather than a sharp transition as previously believed. The effects of anionicity, *s-d* band hybridization and long range interactions on the dimensional transition are quantified by using the structures adjacent to the minima. Anionicity marginally changes the relative stability of the clusters. The degree of *s-d* hybridization is varied via changing the Hubbard U value which corroborate that *s-d* hybridization alone does not stabilize planar structures. van der Waals interactions, on the other hand, stabilize globular structures. These results elucidate the balance between the different reasons of the dimensional transition in gold nanoclusters.

Gold catalysts have been the subject of intense research after the demonstration of catalytic activity of gold nanoparticles for CO oxidation a few decades ago^1^. As interest grew in gold nanoparticles, other catalytic properties^2^ along with their applications in optoelectronics^3^, molecular assembly^4^, biorecognition and chemical sensors^5^ emerged. From an engineering point of view, the control and manipulation of the properties of nanoclusters in these applications are critical. Two important control parameters in nanoclusters are size and shape^6^,^7^. Considerable effort has been devoted to gold clusters with fewer than 100 atoms to understand the geometric properties of various sizes, and as a result several interesting observations have been made: in contrast to alkali and other noble metal clusters which exhibit planar (2D) to globular (3D) transformations in the size range of 5–7 atoms^8^,^9^, planarity is conserved until the 12–14 atom range for Au nanoparticles^10^,^11^,^12^,^13^. This anomalously high atom number for planar nanostructures is often claimed to be associated with the tug of war between strong 5*d*-6*s* orbital hybridization due to relativistic effects^14^,^15^ and “aurophilicity”^16^, which is broadly defined as an attractive interaction of dispersive character. The structures of the 3D clusters also vary depending on their size. Above the 2D to 3D transformation point, hollow cages, tubes and space-filling (such as tetrahedron, icosahedron, Wulff shaped) structures may form without distinct transition ranges for Au_*n*_ clusters with *n* < 100^13^,^17^,^18^,^19^,^20^.

In determining Au_*n*_ structures, computational approaches^9^,^15^,^18^,^21^,^22^ are heavily employed in addition to spectroscopic measurements^10^,^17^. Among theoretical approaches, density functional theory (DFT) is widely employed since it offers a powerful method for the first-principles prediction of electronic and structural properties of materials at a reasonable computational cost. Using a local minimization scheme such as the conjugate gradient method, the local minimum close to a certain configuration can usually be correctly identified using DFT-derived forces. However, finding the global minimum structure for a given composition requires global minimization techniques such as simulated annealing^23^, genetic algorithm (GA)^24^, basin^21^ and minima hopping^25^, and particle swarm^26^ methods, coupled with a reliable local optimizer. These stochastic global optimization methods have increasingly been utilized in materials discovery and development^27^,^28^,^29^,^30^,^31^,^32^.

GA is an evolutionary algorithm, which is based on the idea of the survival of the fittest. Inspired from nature, the algorithm starts with a set of parent configurations, which is defined as the population. For structural optimization of materials, the parents may have different lattice types, atomic bases and even composition. Some of the individuals in this population are more fit – which in the context of structural optimization means lower in total energy – based on their structures compared to others. Fit structures survive and are allowed to mate. Mating is accomplished by crossing patterns (genes) between fit individuals. During crossover, random mutations in the genes are also allowed to a certain degree to avoid a stagnant gene pool and a better sampling of the phase space. The offspring individuals form the next generation of parents and this process continues until some pre-defined criteria are met. In creating the genome of a structure, two methods are generally applied. In the first one, the properties of the structure, such as lattice parameters, lattice angles, location of atoms, type of atoms *etc*. are converted to bit-strings^33^ which are subjected to crossover. In the second method, two “fit” parent structures are spatially decomposed into smaller fragments. Then, the fragments from different parents are combined to form a child structure, mimicking the process of dividing and recombining chromosome pairs in biology^34^.

Gold nanoparticles have been studied using global optimization algorithms. Basin hopping, which is reminiscent of a Monte Carlo method, combined with an empirical potential (for Au_*n<*110_)^21^ and DFT calculations (for Au_*n*<20_)^18^ have been used to study unsupported clusters. Supported Au_8_ on MgO was investigated with a genetic algorithm coupled with DFT^35^. These studies, however, did not discuss or provide an ensemble of low energy structures that were close enough in energy to the global minimum to be accessible during typical synthesis conditions. Charge and compositional effects on 2D–3D transition in 8 atom AuAg bimetal clusters have also been studied with DFT-GA approach^27^,^36^. It is shown that above 25% Ag alloying in neutral gold clusters results in transition from planar to globular^27^. For anions (cations) 2D–3D transition occurs in silver (gold) rich regime^36^. Yet, the impacts of ionicity and long-range interactions on the relative stabilities of near minima have not been reported in the Au_12–14_ size range.

In this study, we implement a genetic algorithm employing 4-parent crossover and adaptive genetic manipulations and couple it with DFT to search for global minima in isolated Au_12_, Au_13_, and Au_14_ clusters. Both 2D and 3D evolutions are pursued, *i.e.* with and without restricting the atoms to planar geometries, for each cluster size. Our goals are to obtain the ground state structures in 2D and 3D and generate a collection of near-ground state isomers that could be observed due to thermal excitations or non-equilibrium synthesis techniques. We also investigate the relationships between planarity and total energies from the large set of structures generated from the global optimization for Au_12–14_. We test our GA-DFT approach on anionic clusters to identify the impact of an excess electron in the near minima energy landscape. Within the context of neutral clusters, we discuss the effect of *s-d* orbital hybridization, spin-orbit coupling and long-range interactions on the 2D–3D transition.

## Results and Discussion

### Neutral Clusters

**Figure 1:**
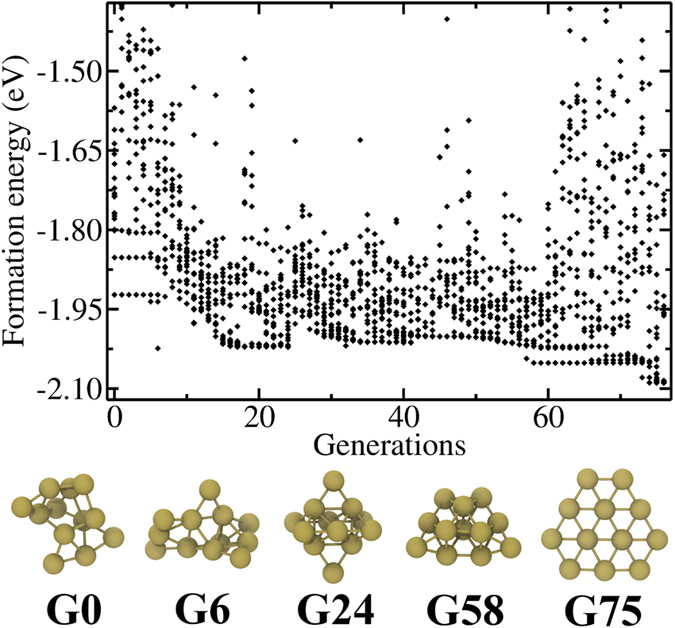
Per atom formation energies and structures of 3D Au_12_ clusters as a function of generation number through GA optimization. The lowest energy clusters at selected generations (given by G#) are also presented in the bottom panel.

**Figure 2:**
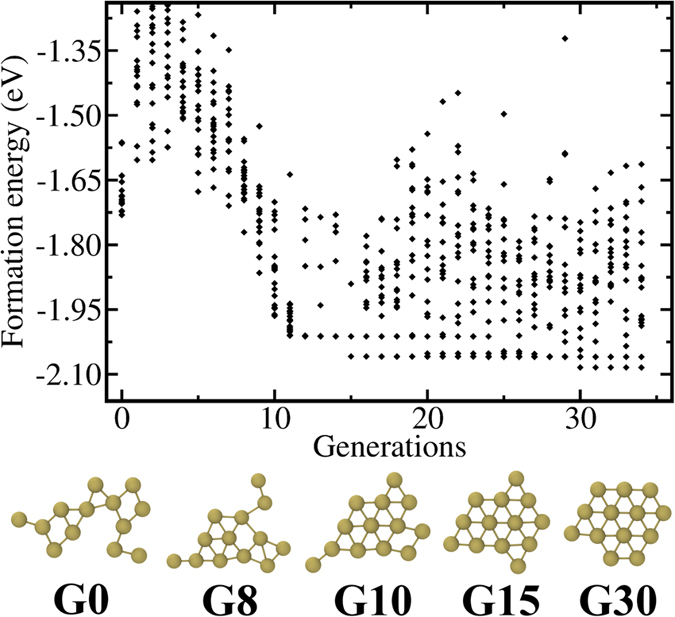
Per atom formation energies and structures of 2D Au_13_ clusters as a function of generation number through GA optimization. The lowest energy clusters at selected generations (given by G#) are also presented in the bottom panel.

**Figure 3:**
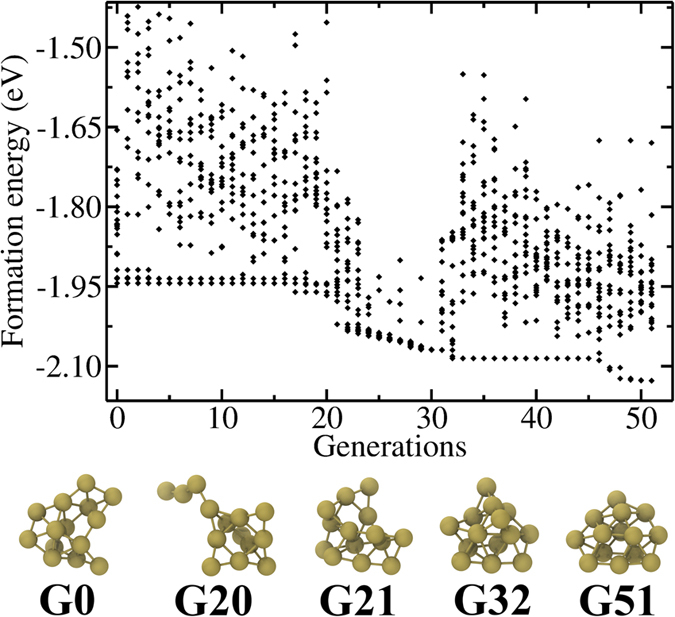
Per atom formation energies and structures of 3D Au_14_ clusters as a function of generation number through GA optimization. The lowest energy clusters at selected generations (given by G#) are also presented in the bottom panel.

The combined GA-DFT algorithm is utilized several times to obtain global minima in 12, 13 and 14 atom planar and globular Au particles. In Figs 1, 2 and 3, we present the evolution for Au_12_, Au_13_ and Au_14_ clusters starting from 3D, 2D and 3D structures as these GA evaluations produce the identified global minima for the corresponding cluster sizes. The total energies of all 20 members of the population are given at each generation, along with lowest energy systems at selected generations, to show the structural evolution and some common structural motifs throughout the optimization process. Similar plots for 2D Au_12_, 3D Au_13_ and 2D Au_14_ are given in Figures S1–S3 in the Supplementary Information (SI). In all these figures, we report the per atom formation energies of the clusters from non-interacting gold atoms (Note that the DFT calculations on the clusters have been performed without spin polarization during GA minimization). The energy of a single gold atom is calculated using the same settings as the clusters except that we also considered spin polarization for the atom. Since the energy differences between spin polarized and non-spin polarized calculations are very small for clusters, we expect the formation energies are reliable. Starting from a randomly constructed (subject to bond length constraints, see Methods) Au_*n*_, the coupled optimization scheme results in energy drops of ~2 to 4.6 eV in about 50 generations. Considering that every generation consists of 20 members, structures at or close to the ground state have been identified in approximately 1000 DFT calculations. Earlier studies that utilize basin hopping and DFT for the prediction of global minima in C-H-O clusters with similar numbers of atoms also required about 1000 DFT calculations^37^. An important advantage of GA, compared to basin hopping, lies in the crossover operations where large distances can be traversed in the phase space and sampling from wide apart phase space volumes can be obtained. In basin hopping, the trial movements for the atoms are not drastic^38^ and resemble the mutations in GA rather than crossover. Such an advantage may explain the efficiency of GA in finding many near-equilibrium structures which were not obtained by the previous basin-hopping search.

For 12- and 13- atom clusters, 3D evolution resulted in the planarization of the initially generated globular geometries as shown in Figs 1 and S2 respectively. The lowest energy structures (G75 in Fig. 1 and G30 in Fig. 2) are the ones that minimize the edge length, simultaneously minimizing the number of dangling bonds and maximizing the average coordination number in the plane. The evolution restricted to 2D produces similar planar geometries for these clusters. The GA predictions for Au_12_ and Au_13_ minima, see Au_12_(1) and Au_13_(1) structures in Tables 1 and 2, are same as the ones given by Lee *et al*.^22^ On the other hand, Au_13_(2) in Table 2 is proposed as the lowest energy structure for Au_13_ in another study which used basin hopping^12^. For the 14-atom system, the lowest energy (G51 in Fig. 3) is obtained for a pouch-like structure which was previously suggested as a candidate for global minimum of anionic clusters^18^. Apart from lowest energy structures, we have identified structures that have energies per atom within 2*k*_B_*T* of the minima at room temperature *T* in each cluster system. Note that we use an increased cutoff energy and consider spin-polarization to re-evaluate these clusters as mentioned in Methods section. These near-minima structures are shown in Tables 1, 2 and 3. The energy differences (Δ*E*) of these structures from the candidate minima are also included per atom basis below each cluster. The Cartesian coordinates of the clusters in these tables are available in xyz format in the Supplementary Dataset (SD). From the tables, it is seen that the 2D structures are generally edge variants of each other. The near-minima include both 2D and 3D clusters, indicating that the transition from 2D to 3D structures is not abrupt for gold clusters in the 12–14 atom range. Accordingly, it is possible to experimentally observe an ensemble of structures mixing 2D and 3D geometries at room temperature.

**Figure 4:**
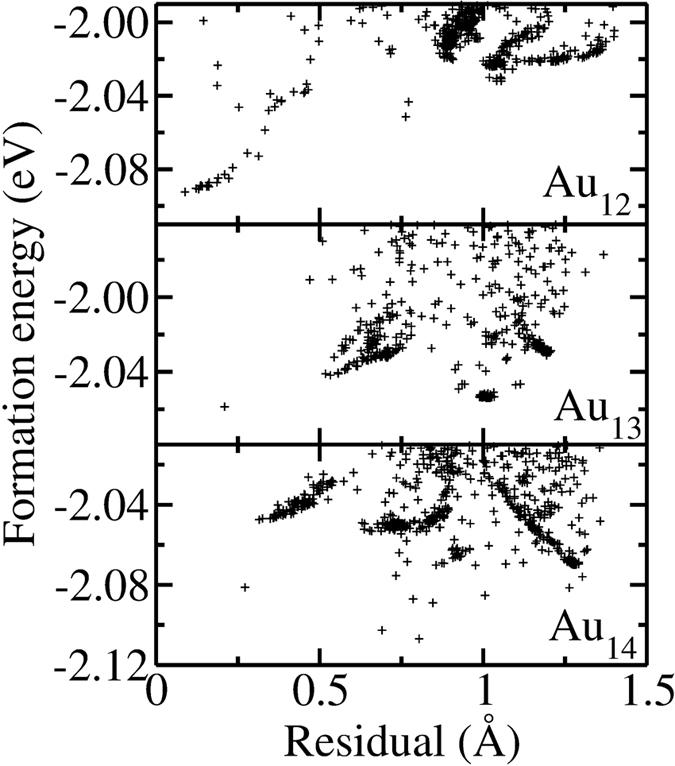
Per atom formation energies of 3D Au_12–14_ clusters as a function of the total deviation of atom positions from the best plane (*i.e.* residual) that represents the corresponding cluster.

The global structural optimization combining GA and DFT is able to predict global minima along with many energetically similar structures near the 2D to 3D transition size. At the same time, we generate thousands of sample clusters from various regions of the energy and coordination landscape. Using these samples, we investigate the relationship between energy and planarity, *i.e.* whether, in general, the 2D structures are favored for Au_12_ and Au_13_ and 3D structures are favored for Au_14_. For each cluster, we locate the best-fit plane with the least squares algorithm. The planarity of the cluster is measured by the sum of normal distances between each atom in the cluster and the fitted plane (*i.e.* residual). A lower residual means that the structure is closer to a planar geometry. In Fig. 4, the formation energies of 3D Au_12–14_ clusters with respect to their residual values are presented. We only include structures that have energies within 100 meV/atom of the lowest energy in this plot. In 12-atom clusters, there seems to be a trend where the energy is lowered by decreasing the residual. Also the energies of the structures that have relatively higher residuals (1–1.5 Å) are considerably higher than suggested global minima. On the other hand, for 13 and 14-atom clusters, a number of clusters with very different degrees of planarity nonetheless have very similar energies. This illustrates the gradual rather than abrupt transition between 2D and 3D structures.

### Anionic Clusters

**Figure 5:**
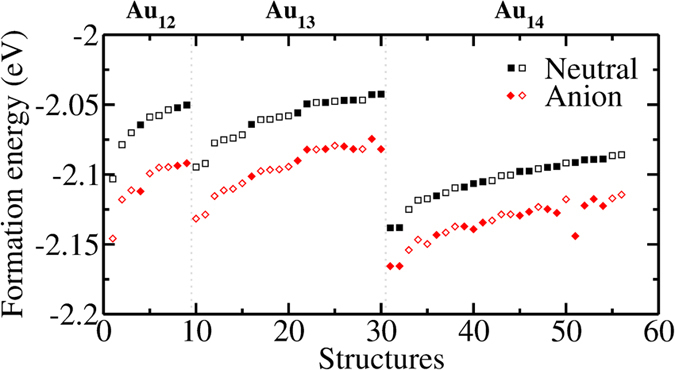
The effect of excess electron on the per atom formation energies of the low energy Au_12–14_ clusters identified in Tables 1, 2 and 3. Formation energies are shown for 56 structures. The clusters are given in the same order as in Tables 1, 2 and 3 for Au_12_ (structures 1–9), Au_13_ (structures 10–30) and Au_14_ (structures 31–56). The vertical dashed lines separate one cluster size from another. Empty and filled symbols are used for 2D and 3D structures respectively.

We also test the consistency of energy ordering between non-spin polarized/neutral and spin-polarized/anionized (Au_13^−^_) calculations, since experimentally reported clusters are generally ionized. For this estimation, we use 1000 randomly generated 2D and 3D clusters. The probability of having the same energy ordering is found to be 96.9% between non-spin polarized/neutral and spin-polarized/anionized clusters. Although the formation energy order seems to be similar to a high degree for the randomly created anionic and neutral clusters, we also check the near-minima by re-evaluating the structures in Tables 1, 2 and 3, and performing four additional GA optimizations for Au_13^−^_. The energy comparison between neutral and anionic clusters is shown in Fig. 5 for structures in Tables 1, 2 and 3. The results of GA optimizations are given in Table S1 in SI and the coordinates of the low energy structures are given in SD. The formation energies of anionic clusters (*E*_*f*_) are calculated as given in Equation 1.


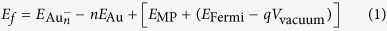


where 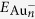 is the total energy of anionic cluster of *n* atoms. *E*_Au_ is the energy of an isolated gold atom. *E*_MP_ is the monopole term of Makov-Payne^39^ correction for the image charge interactions between the extra electron and neutralizing background charge. *E*_Fermi_ and *V*_vacuum_ are the Fermi level and electrostatic potential in the vacuum away from the cluster *q* is the charge of the cluster.

Re-evaluation of near-minima of neutral clusters with an excess electron does not change the energy order drastically. The minima are not altered for 12, 13 and 14-atom clusters in terms of cluster dimensionality and cluster structures. Any large drop in the energy (as in the case of the 51^st^ structure in Fig. 5, Au_14_(21) originally in Table 3) is related to relaxation of the cluster to a new local minimum structure. From the GA optimization of Au_13^−^_, we obtain many structures identical to the ones found for neutral clusters. Anionic form of Au_13_(1) is again the minimum energy structure of this cluster size. In addition to already determined clusters, we identify more than a dozen new 2D and 3D structures that are in 2*nk*_B_*T* (*n* = 13, *T* = 300K) of the minimum as seen in Table S1.

Gas-phase ion mobility measurements of negatively charged gold clusters have been performed by Furche *et al*.^40^.The proposed 12- and 13-atom nanocluster structures are both captured by our GA + DFT calculations as Au_12_(1) and Au_12_(4) in Table 1 and Au_13_(3) and Au_13^−^_ (5) in Table 2 and Table S1 respectively. High resolution photoelectron spectroscopy^11^ and trapped ion electron diffraction^13^ measurements have also been conducted for anionic Au clusters. The candidate isomers that show reasonable fit to experimental data are also captured by the GA + DFT approach presented here. For Au_12^−^_ and Au_13^−^_, the same nanocluster structures are predicted as in ref. 40. For Au_14^−^_, the presented structure corresponds with Au_14_(1) in Table 3.

### Relativistic Effects and *s*-*d* hybridization

One of the unusual properties of Au_*n*_ clusters is that the transformation from planar to globular structures occurs at larger sizes (*n* = 12–14) compared to other metal clusters such as Cu_*n*_ and Ag_*n*_ (*n* = 6–7). A proposed reason for this observation is the enhanced 5*d*-6*s* hybridization due to relativistic effects in gold favoring the 2D structures at larger cluster sizes^15^. Our GA-DFT calculations also predict a 2D–3D transformation at large cluster sizes (i.e. after Au_13_). In order to investigate the structure-hybridization relationship in this transition range, we calculate the *s*-*d* hybridization in both 2D and 3D clusters. In estimating the degree of hybridization (*H*_*sd*_), we used two definitions: In the first, shown in Equation 2, the common area under the *s* and *d* projections of density of states (DOS), namely *g*_*s*_ and *g*_*d*_, are calculated up to Fermi energy (*E*_*F*_).


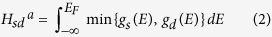


In the second more rigorous approach, the degree of *s-d* hybridization is found by multiplying the local *s* and *d* orbital projection of each Kohn-Sham eigenstate (*w*_*s*_ and *w*_*d*_)^15^, see Equation 3.


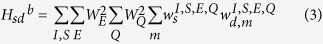


**Figure 6:**
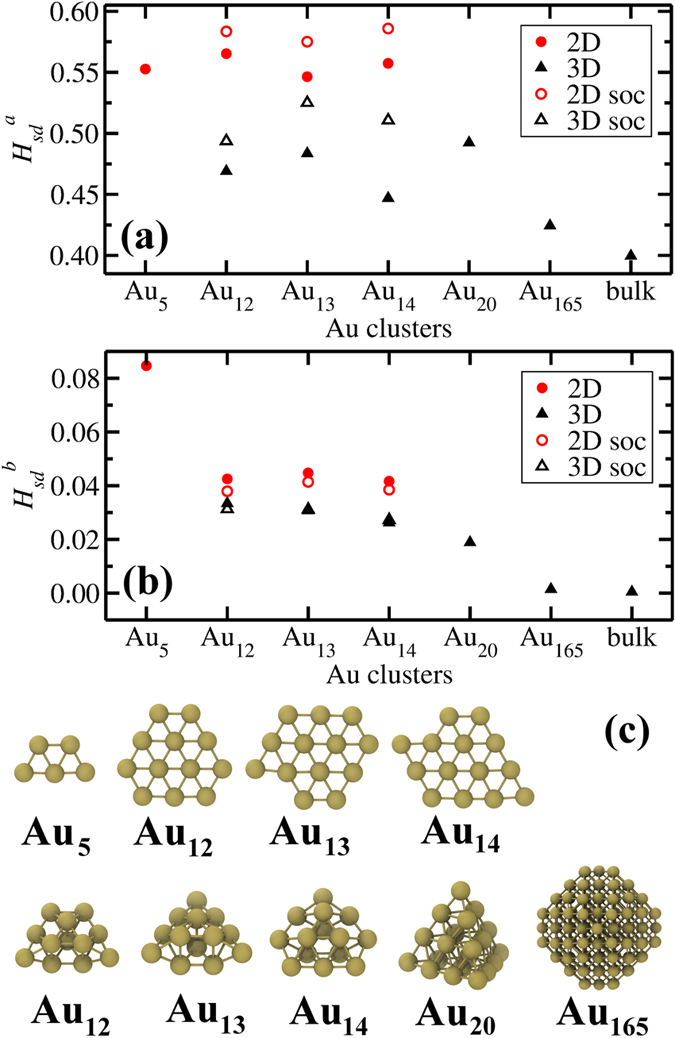
*s-d* band hybridization index, *H*_*sd*_, defined as (**a**) the common area under *s* and *d* shell decomposed electronic density of states and (**b**) the product of *s* and *d* weights of local charges for different sized Au clusters in 2D and 3D constructions. Spin-orbit coupling (soc) is also considered in the case of Au_12–14_ clusters. (**c**) The atomic structures of the evaluated particles.

The weights *w*_*s*_ and *w*_*d*_ are obtained from the projection of total wavefunction onto spherical harmonics within a sphere around each atom^41^. They are calculated at each reciprocal space point *Q*, band energy eigenvalue *E*, spin component *S* and atom *I* so summation over all these are needed. The *m* (*i.e.* projected angular momentum component) summation is only relevant for the *d* orbitals. *W*_*E*_ and *W*_*Q*_ are band occupancy and weight of the reciprocal space point. In Fig. 6a–c, we show *H*_*sd*_^*a*^and *H*_*sd*_^*b*^ per atom and evaluated clusters respectively. The structures for Au_12–14_ are obtained from the GA optimizations. For comparison, we include the same *s-d* hybridization indices for Au_5_, Au_20_ structures taken from literature^11^,^17^, a Au_165_ nanocrystal created from the Wulff construction from (111), (100) and (110) surface energies^42^, and bulk Au.

In Fig. 6a, *H*_*sd*_^*a*^ is presented for the 2D 5-, 12-, 13-, 14-atom and 3D 12-, 13-, 14-, 20-, 165-atom clusters and face centered cubic (bulk) gold. The planar structures assume very similar values for this measure of hybridization. 3D forms consistently have lower values of *H*_*sd*_^*a*^ compared to planar ones. *H*_*sd*_^*b*^ also shows that 2D clusters possess higher numbers of electrons in *s-d* hybrid orbitals, see Fig. 6b. Close-packed FCC gold has the smallest value in both measures. The odd numbered clusters, except for Au_165_, are found to have non-zero magnetic moments and the even numbered ones are non-magnetic. Au_165_ is large enough that there are many 12-fold coordinated atoms, so one may expect Au_165_ to behave bulk-like. Evidently, its *s-d* hybridization index is close to that of bulk Au. In particular, we note that across the transition range of 12–14 atoms, the *s-d* hybridization indices remain constant for the 2D structures and decrease as the structures become more compact (*i.e.* going from pouch-like to face-centered close packed structures) for the 3D structures. This indicates that the energy contribution due to hybridization is more or less the same for the 2D minima. The total energy, on the other hand, increasingly favors 3D structures as the size increases. In order to investigate and quantify the energetic influence of *s*-*d* hybridization, we artificially change, for the lowest energy 2D and 3D structures at *n* = 12–14 given in Fig. 6, the amount of *s-d* hybridization by shifting the energies of the *d*-bands *via* a Hubbard *U* correction with *U* = 0.25–4 eV. We find that increasing *U* leads to a decrease in *H*_*sd*_^*a*^ and *H*_*sd*_^*b*^ (except for *H*_*sd*_^*b*^ of 3D Au_14_) in both planar and globular clusters as seen in Figure S4 in SI. The decrease is more pronounced for 2D than 3D structures for *n* = 12–14. If *s*-*d* hybridization were responsible for stabilizing the planar structures, a more pronounced decrease in *s-d* hybridization in the 2D structures should lead them to become less stable *vis a vis* the 3D structures. But in fact, at *n* = 12–13, 2D structures are further stabilized relative to the 3D structures by about 5–6 meV/atom. The opposite trend is seen at n = 14 but only marginally (~1.5 meV/atom). This evidence suggests that *s-d* hybridization is not directly correlated with the stabilization of 2D structures. This is consistent with the earlier studies on Au_8_ clusters emphasizing that large *s-d* hybridization does not necessarily mean high stability for planar structures^43^.

For a rigorous description of hybridization, relativistic effects should be considered in the calculations^15^. The relativistic contraction of valence n*s* shells and expansion of (n-1)*d* shells cause the overlap of these states and hybridization^16^. Our DFT calculations include scalar relativistic effects (*i.e.* Darwin and mass-velocity terms). For a more complete analysis of relativistic effects, we also consider spin-orbit coupling for Au_12_, Au_13_ and Au_14_ in both planar and 3D forms. The results of these computations are shown in Fig. 6. Again, 2D structures show a higher hybridization index compared to 3D constructions. A shift in the absolute values of the *H*_*sd*_ is observed in spin-orbit coupling compared to scalar relativistic calculations. However, the differences between planar and globular structures are almost unchanged. These results point that the correct behavior in 2D–3D transition range can be captured for gold clusters with scalar relativistic calculations and addition of spin-orbit coupling does not change the hybridization behavior significantly.

### Long Range Interactions

**Figure 7:**
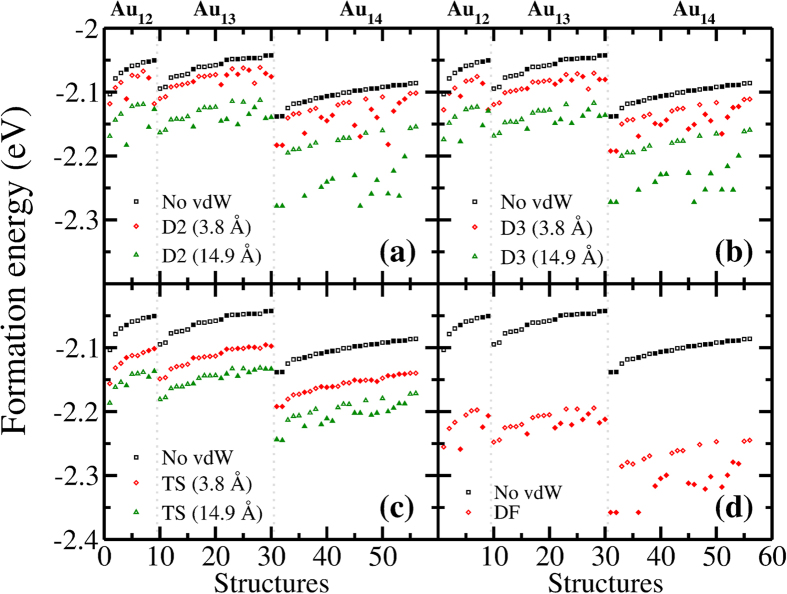
The effect of (**a**) D2, (**b**) D3, (**c**) TS and (**d**) DF vdW interactions on the per atom formation energies of the low energy Au_12–14_ clusters identified in Tables 1, 2 and 3. Formation energies for different interaction cutoffs, which are given in parenthesis, are shown for 56 structures. The clusters are given in the same order as in Tables 1, 2 and 3 for Au_12_ (structures 1–9), Au_13_ (structures 10–30) and Au_14_ (structures 31–56). The vertical dashed lines separate one cluster size from another. Empty and filled symbols are used for 2D and 3D structures respectively.

Up to this point, the calculations have not involved long-range correlations. Rehr *et al*.^44^, using perturbation theory, estimated the contribution of dipole-dipole and higher order polarization energy to be 17% of the cohesive energy in metallic gold. These interactions are analogous to van der Waals attraction. Accordingly, we first compare different flavors of DFT van der Waals (vdW) correction methods, i.e. D2^45^,^46^, D3^47^, TS^48^, and DF^49^,^50^, for their contribution to the cohesive energy of bulk Au. We find that the energy contribution of the vdW corrections, defined as |ΔE_vdW_ - ΔE_PBE_|/ΔE_vdW_ (ΔE_vdW/PBE_ is the formation energy of the cluster calculated with/without vdW corrections), increases in the order of TS, DF, D3 and D2, giving approximately 12%, 16%, 18% and 19% of the formation energy, when the interaction cutoffs are selected sufficiently large (see Table S2 in SI). The calculated contributions to cohesive energies for the given vdW approximations are reasonably close to earlier estimation of by Rehr *et al*.^44^, but the range from 12% to 19% gives us an opportunity to investigate the effect of varying vdW interaction strengths on the 2D-to-3D transition. In Fig. 7a–d, the change in per atom formation energies, compared to PBE without vdW corrections, of the clusters presented in Tables 1, 2 and 3 are shown for different vdW approximations. We use two vdW interaction cutoffs, 3.8 and 14.9 Å for D2, D3 and TS. The former cutoff only includes the first nearest neighbors and the latter includes all cluster atoms. In the case of DF, a self-consistent solution is obtained for the entire cluster, thus its effect on formation energy is similar to empirical approximations at large cutoffs. The calculations considering only the nearest neighbors reduce the energy of the globular structures but not enough to change the minima for 12 and 13-atom clusters. For both cutoff values, it is seen from Fig. 7 that D2 gives the strongest and TS gives the weakest contribution to the formation energy as the former reduces the energy of 3D structures the most and the latter the least. For the TS calculations, the global minima of Au_12–14_ have not been changed. When D2, D3 and DF methods are utilized, the energy of a globular structure, namely Au_12_(4), is lowered below the planar structure (14 meV for D2, 3 meV for D3 and 3 meV for DF in per atom formation energy). In Au_13_, none of the globular structures, which are shown in Table 2, is reduced in energy below planar ones. However, since the energetic order has changed in Au_12_ to within few meV, one might expect a similar situation for Au_13_. As it turns out, one of the globular structures that are found during GA optimization of 13-atom anionic clusters is actually lower in energy than planar Au_13_ when vdW interactions are considered in the neutral form. This cluster is given in Table S1 in the SI as Au_13^−^_(5). For the D2, D3 and DF methods, the energy difference between this structure and Au_13_(1) is −10 to −17 meV per atom. When the strengths of vdW contributions to the cohesive energy of bulk gold are considered, the D3 and DF methods give the closest approximations to the estimations in ref. 44. The DF method is also shown to closely reproduce highly accurate results from quantum Monte Carlo and coupled cluster calculations^51^. Therefore, when appropriate dispersive interactions are included, we find that the 2D–3D transformation occurs at lower values of *n*, i.e. Au_12_ or Au_13_ rather than Au_14_. However, whether dispersive interactions are included or not, at the transition cluster sizes, 2D and 3D structures coexist within the vicinity of *nk*_B_*T* (*T* = 300 K), showing that the transition is still a gradual one around room temperature.

## Conclusion

We introduce a genetic algorithm – density functional theory method for prediction of stable structures of clusters, and apply it successfully to free-standing Au_12–14_ nanoclusters. The method is capable of not only finding global minimum, but numerous local minima that will assist the experimental characterization of the synthesized clusters and further computational studies concerning catalytic and photonic properties. The GA optimization is based on physically dividing and recombining clusters, as opposed to bit manipulations that are, in some cases, used to find global minimum in periodic systems. The developed GA code can utilize as many parents as possible, with the only condition that each parent should contribute at least one atom. This helps in keeping the gene pool dynamic. Considering the size of the clusters, four parents are used for crossover in this study. This method is found to outperform two parent crossover or random selection for the systems under study. For the mutations, we use a situation-dependent scheme. The lowest energy structures for 12- and 13- atom neutral clusters are found to have planar geometries whereas a 3D form is obtained as the lowest energy for Au_14_ when no long-range interactions are considered. When D2, D3 and DF flavors of van der Waals interactions are included in the energy calculations, the 2D–3D transition size is reduced to below 13 atoms. The strength of dispersive interactions is found to be weaker for the TS van der Waals interaction without appreciably affecting the stability order. It is also shown that the effect of 5*d*-6*s* hybridization can be predicted by scalar relativistic calculations and the inclusion of spin-orbit coupling in the calculations did not significantly change the difference in hybridization indices between planar and globular clusters. The planar structures are found to have higher hybridization index compared to globular clusters for all sizes, and a decrease in the amount of *s*-*d* hybridization in 2D structures did not consistently correlate with energetic destabilization, leading us to conclude that *s*-*d* hybridization is not a significant factor in 2D–3D transition. When the clusters are ionized, the minima configurations are not altered and formation energy order between clusters is mostly unchanged. In all these calculations, we find several dozen clusters with energies that are in close proximity to the lowest energy structures. We predict that these lowest energy and near-lowest energy structures are likely to coexist at room temperature and above due to thermal excitations. The existence of many structures within a small energy interval may explain the long standing debate on the transition size and the global minima of the gold clusters around the studied size range.

## Methods

The atomic configurations of gold clusters are optimized by combining GA and DFT calculations. The GA provides a non-local sampling of cluster structures over the phase space using genetic operations, and the DFT calculations are used for local optimization and total energy calculations.

### Genetic Algorithm

**Figure 8:**
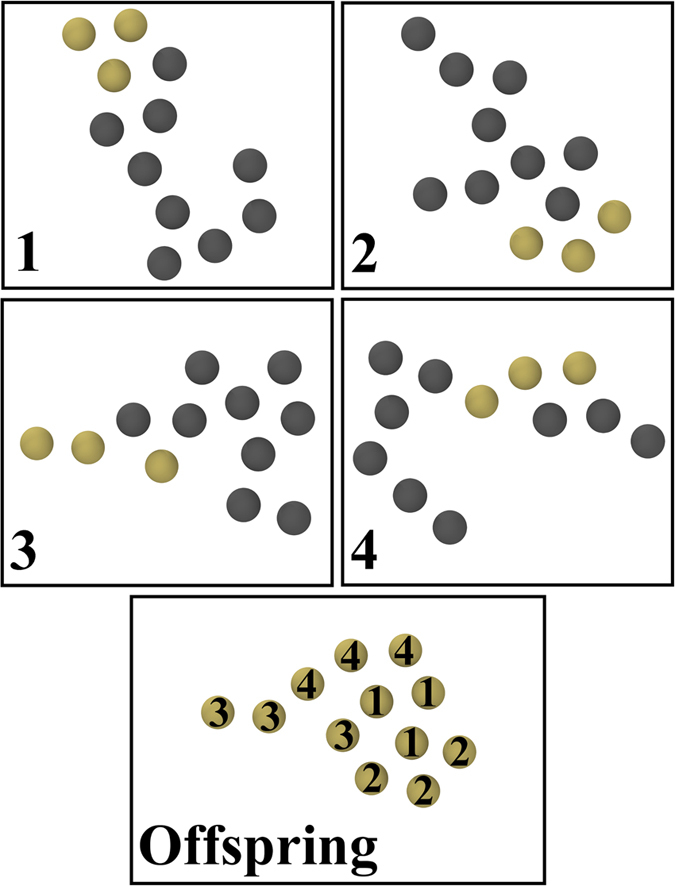
4-parent crossover scheme for a 2D gold cluster. The first 4 panes represent the parents in which the contributed genes are yellow colored. The last pane shows the offspring which is formed from the combination of the selected atom groups from the parents.

We developed a GA code that is distinct from existing GA codes primarily in that it uses a spatial decomposition scheme for crossover with 4 parents, as shown in Fig. 8. Furthermore, the mutation scheme and rate are adjusted automatically during the optimization. Spatial decomposition is realized by dividing each cluster (*i.e.* parent) into four parts, each of which is a connected subcluster of atoms, using either planar or irregular cutting of the parent structure. The mating process involves taking these subclusters from four parents and recombining them into a new cluster. Other genetic operations such as mutations, parent exchange (*i.e.* introduction of randomly generated parents in place of existing ones), and parent cloning (*i.e.* fit parents may be copied to next generation subject to only mutation) are included to increase the optimization speed and prevent premature convergence of the process. For mutations, two operations are considered: (1) A bulk mutation where a randomly selected atom is moved along a random vector within a certain sphere around the mass center of the cluster, (2) an edge/surface mutation where an atom (at position 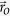), chosen among the ones having the lowest coordination numbers, is moved along the edge/surface. A surface mutation vector is determined on a plane with normal (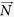) defined by adding the vectors from nearest neighbors to the mutating atom, weighted by the inverse distance, *i.e.* 
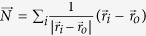
, where the index *i* runs over all nearest neighbors. The surface mutation vector is selected inside a radius of 1.5 bond lengths on the plane defined by 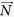. Consequently, the mutating atom moves to a lower density region at the surface. Bulk mutations are applied during the initial generations where the energy differences between structures are large. Edge/surface mutations, on the other hand, are considered when similar structures start to dominate the population. The mutation rate is set to 20% initially but is adjusted on the fly depending on the stagnancy of the lowest energy during the evolution. The population size is selected as 20 and at each generation, 10 structures with the lowest DFT energies are selected for mating operations. For the initial generation, structures are generated randomly subject only to limits on the largest (20 Å) and smallest (2.4 Å) distance between any two gold atoms, in order to give reasonable and sufficiently varied initial guesses for cluster size and atomic bond length.

**Figure 9:**
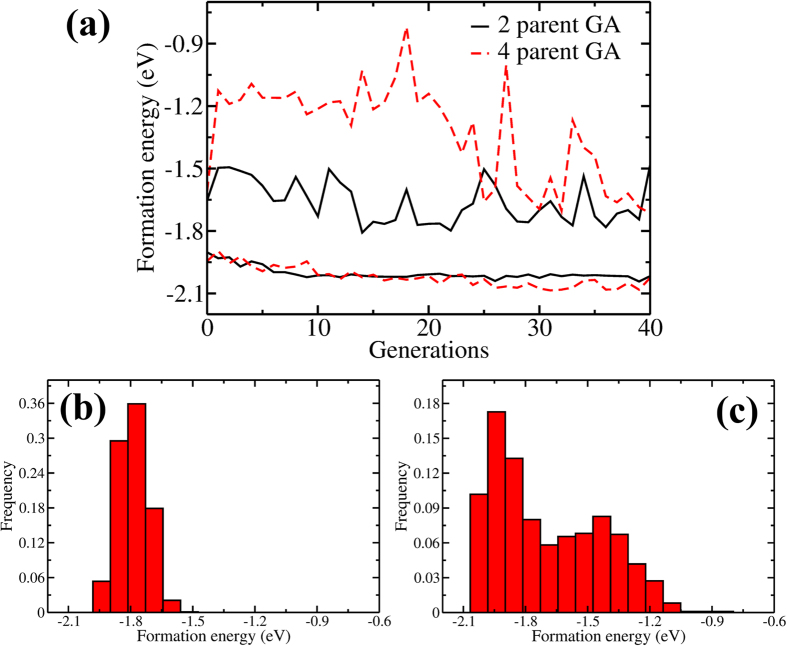
(**a**) The upper and lower limits of the Au_13_ formation energies at each generation during GA optimization with 2-parent (black solid lines) and 4-parent (red dashed lines) crossover schemes. A population of 20 is used for both schemes. Comparison between the energy sampling of (**b**) randomly-generated and (**c**) GA-generated Au_13_ nanoclusters. The data is based on 1000 randomly generated clusters for (**b**) and the first 1000 structures obtained from a 4-parent GA optimization (i.e. 20 clusters over 50 generations) for (**c**). Per atom formation energies are given for all graphs.

We evaluate the energy distribution of structures obtained from GA sampling to report its merits in adequately covering the phase space. In this respect, we first compare the 4-parent versus 2-parent crossover GA schemes. For each type of crossover, we perform 4 independent GA optimizations with a population of 20, lasting 40 generations. When the entire sampling from these calculations is investigated, it is seen that 4-parent crossover scheme samples a larger energy space and produces lower energy structures, as seen in Fig. 9a. We also compare the energy distribution obtained from GA with that obtained from random sampling. The same number of ionic relaxations (*i.e.* 18 conjugate gradient steps) is considered for structures created by both sampling methods. In Fig. 9b, we show the energy distribution of random sampling and in Fig. 9c, the energy distribution from a GA optimization run. It is seen that the randomly generated clusters produce a Gaussian-like distribution with certain energies sampled much more than the rest. The results in Fig. 9c show that GA samples a larger energy space extending both to lower and higher energies in a more uniform fashion. This is practically important if the DFT results are to be used as training set for the parameterization of empirical potentials^52^. It is true that the random sampling can be further extended to higher energies by widening the range of accepted atom-atom separations and the distribution can be balanced by careful selection of structures from the randomly generated samples. However, it is much harder to create sufficient number of low energy structures with random generation and any parameterization of empirical methods with a randomly generated training set will be deficient in predictions around ground state.

### DFT

First principles calculations on clusters are performed with a plane wave basis as implemented in the DFT code VASP^53^,^54^. The projector augmented wave (PAW) method is used for efficient description of valence states near the cores. The Perdew-Burke-Ernzerhof (PBE)^55^ parameterization of the generalized gradient approximation (GGA) is selected for electron exchange-correlation since this functional is found to perform well for nanoclusters^56^,^57^. In fact, PBE is shown to reproduce the CCSD(T) (coupled cluster with full single/double excitations and many-body perturbation theory estimate of triple excitation) and B2PLYP (doubly hybrid functional with perturbation corrections) results for the structure of Au_8_^56^. When the vdW-DF method is used for the dispersion interactions, the optB86b-vdW functional is employed. After testing the energy convergence and relative stability of clusters (see Figure S5 in SI and the discussion therein), we determine that a plane-wave energy cutoff of 230 eV converges the energy to 7.5 meV per atom with little to no change in relative stability of gold clusters. Therefore this cutoff is adequate for the DFT calculations during the GA optimization. For 2D clusters, we use a simulation box with the dimensions 30 × 30 × 15 Å^3^, whereas for 3D clusters a 30 × 30 × 30 Å^3^ box is used, in order to minimize spurious cluster-cluster interactions through periodic boundary conditions. Additional information on the convergence of energy and simulation box size is given in SI. In all DFT calculations, only the Γ-point is used in reciprocal space. DFT ionic relaxations during the GA run are performed partially (*i.e.* only 18 ionic steps) using conjugate gradient minimization. Partial relaxation is useful in balancing the number of local and global minimization steps and reducing the computational cost. Also, partially relaxed structures ensure a more inclusive sampling around local minima.

To further reduce computational cost, non-spin-polarized DFT computations are performed during GA optimization. We compare the results of spin-polarized and non-spin-polarized calculations on 1000 randomly-generated Au_13_ structures. The total energies of spin-polarized evaluations are found to be on the average 15 meV lower than the non-spin-polarized results, but the energy order between different structures is largely unaffected. The probability of two clusters having the same order in terms of energy with spin-polarized and non-spin-polarized calculations is found to be 99.7%.

One of our aims is to capture Au_12–14_ clusters that are energetically relevant under typical synthesis conditions. Small cluster production techniques such as laser vaporization^58^ are highly non-equilibrium and expose nanoclusters to a range of temperatures. We report all Au_*n*_ clusters that are within 2*nk*_B_*T* (*n* = number of atoms, *T* = 300 K) from the global minimum energy structures. To identify these clusters, after the GA calculations are completed, we further relax the structures within 3*nk*_B_*T* of the predicted minima, using an increased energy cutoff (300 eV) and spin-polarization, until the energy difference between two ionic relaxation steps are converged to 10^−4^ eV/atom. We select structures from 3*nk*_B_*T* proximity because during GA optimization the structures are only partially relaxed and full relaxation can bring some structures below the 2*nk*_B_*T* cap. For these calculations we use a 30 × 30 × 30 Å^3^ simulation box regardless of the dimensionality of the cluster.

## Additional Information

**How to cite this article**: Kinaci, A. *et al*. Unraveling the Planar-Globular Transition in Gold Nanoclusters through Evolutionary Search. *Sci. Rep.* **6**, 34974; doi: 10.1038/srep34974 (2016).

**Publisher's note:** Springer Nature remains neutral with regard to jurisdictional claims in published maps and institutional affiliations.

## Supplementary Material

Supplementary Information

Supplementary Dataset

## Figures and Tables

**Table 1:**
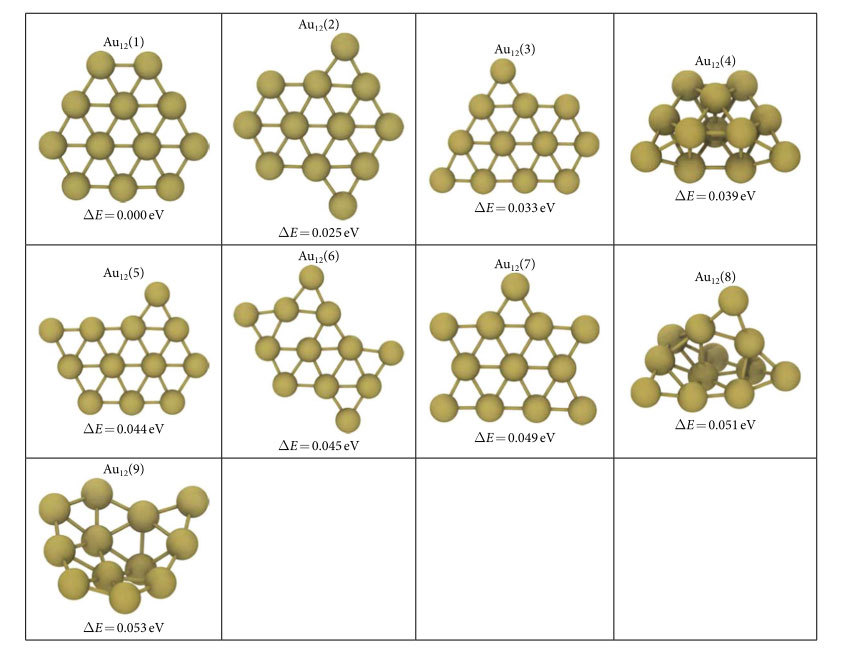
Au_12_ clusters that are in 2*nk*_B_*T* (*n* = 12, *T* = 300 K) proximity of the predicted minimum energy structure.

The quantity Δ*E* is described as the per atom energy difference of the corresponding structure from the minimum which is given as Au_12_(1). See SD file for atomic coordinates.

**Table 2:**
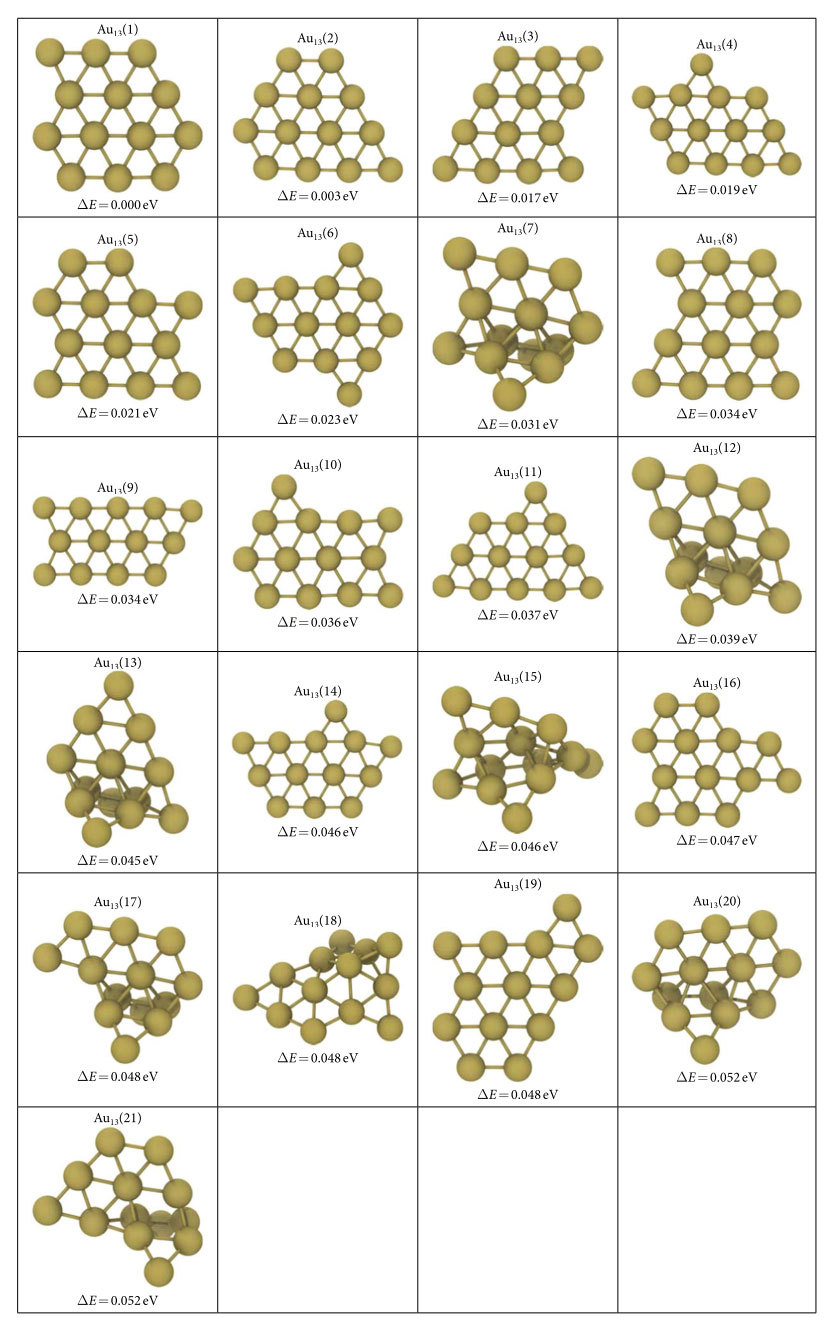
Au_13_ clusters that are in 2*nk*_B_*T* (*n* = 13, *T* = 300 K) proximity of the predicted minimum energy structure.

See SD file for atomic coordinates.

**Table 3:**
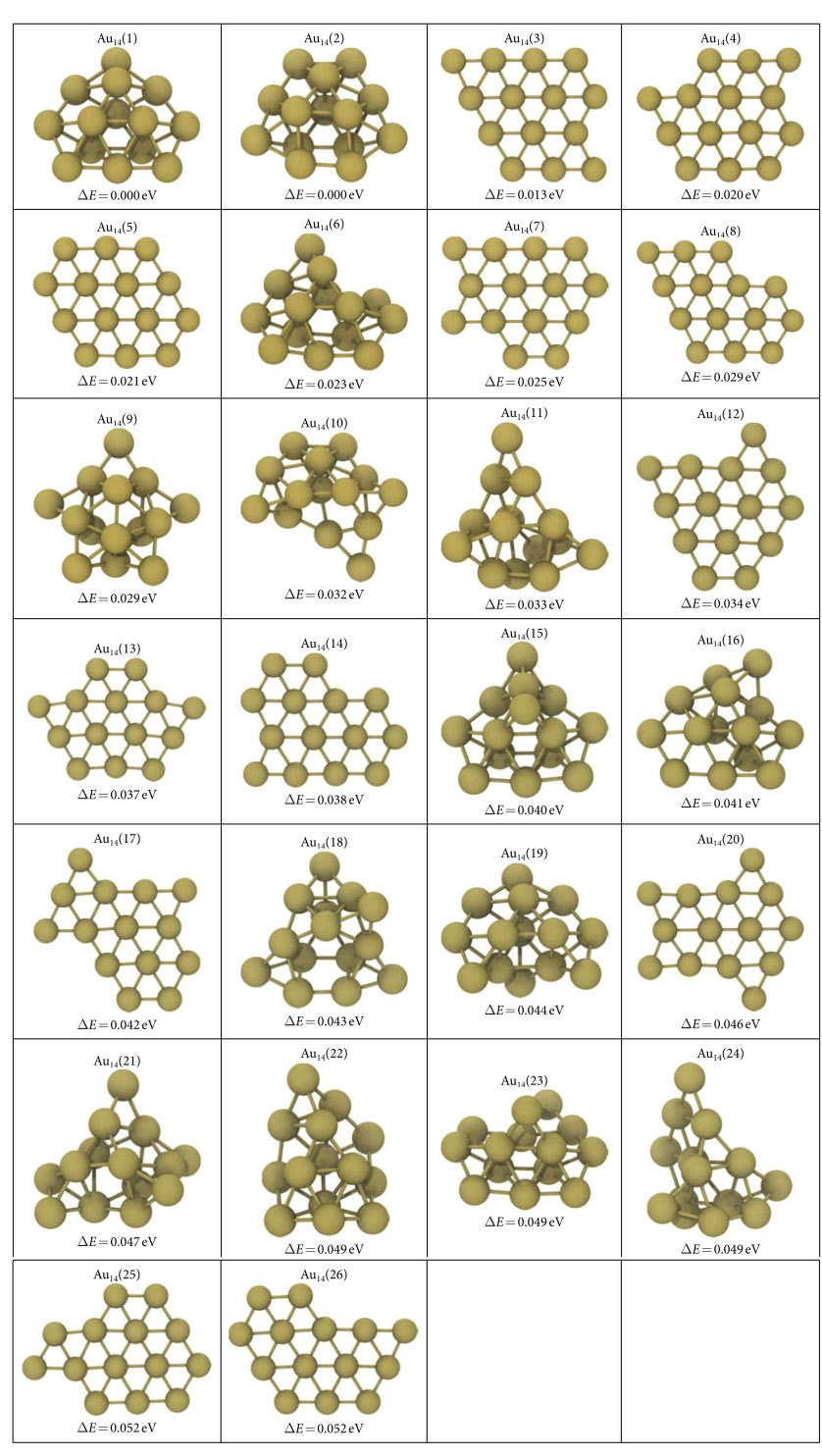
Au_14_ clusters that are in 2*nk*_B_*T* (*n* = 14, *T* = 300 K) proximity of the predicted minimum energy structure.

See SD file for atomic coordinates.
