# Supplementary material for: Unraveling the Planar-Globular Transition in Gold Nanoclusters through Evolutionary Search
Source: Sci Rep. 2016 Nov 28;6:34974. doi: 10.1038/srep34974 (PMC5124999; doi:10.1038/srep34974)
Supplement: Supplementary Information [file srep34974-s1.pdf]

Supplementary Information

Unraveling the Planar-Globular Transition in Gold Nanoclusters through Evolutionary Search

Alper Kinaci,[1] Badri Narayanan,[1] Fatih G. Sen,[1] Michael J. Davis,[2] Stephen K. Gray,[1] Subramanian K. R. S. Sankaranarayanan,[1] Maria K. Y. Chan[1]*

[1]Center for Nanoscale Materials, Argonne National Laboratory, Lemont, IL 60439 USA

[2]Chemical Sciences Division, Argonne National Laboratory, Lemont, IL 60439 USA

**Correspondence and requests for materials should be addressed to M.C. (email: mchan@anl.gov)*

GA optimization of neutral 2D Au_{12}, 3D Au_{13} and 2D Au_{14}

Structural evolution of 2D Au_{12}, 3D Au_{13} and 2D Au_{14} are presented in Figures S1, S2 and S3 respectively. These figures include the per atom formation energies of all evaluated members at each generation and some selected structures that have the lowest energies at the corresponding generations from the evolution path. In calculation of formation energies, single gold atom in vacuum is used as the reference state.

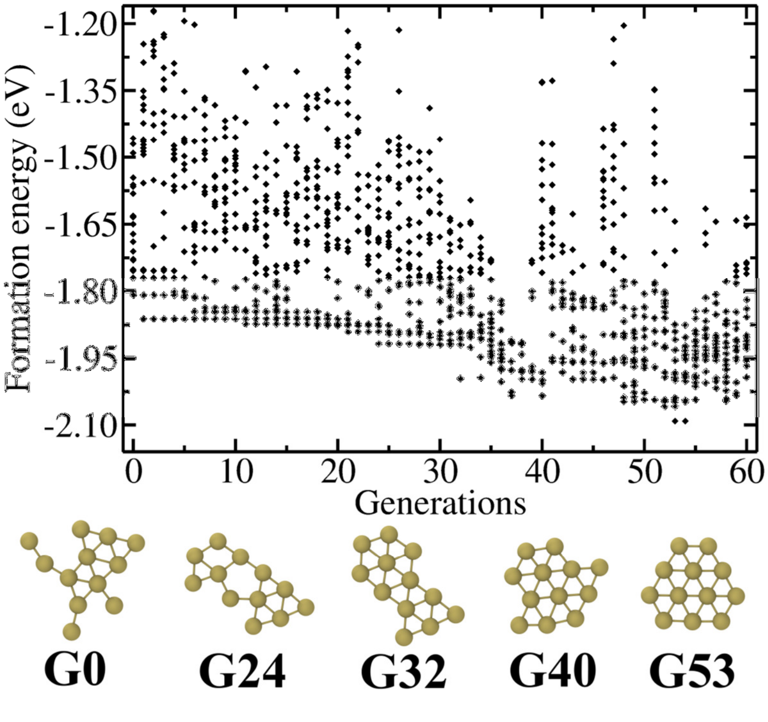

Figure S1: Per atom formation energies and structures of 2D Au_{12} clusters as a function of generation number through genetic algorithm optimization. The lowest energy clusters at selected generations (given by G#) are also presented in the bottom panel.

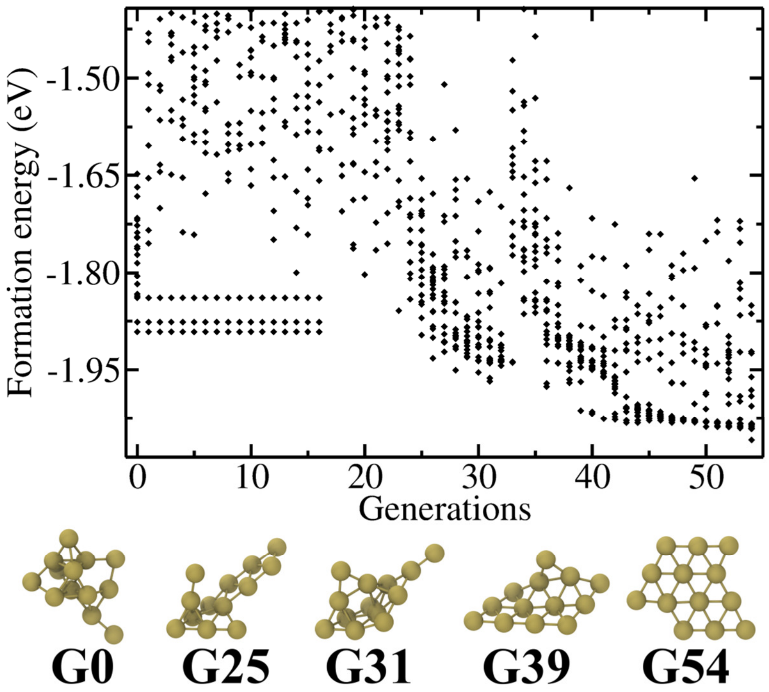

Figure S2: Per atom formation energies and structures of 3D Au_{13} clusters as a function of generation number through genetic algorithm optimization. The lowest energy clusters at selected generations (given by G#) are also presented in the bottom panel.

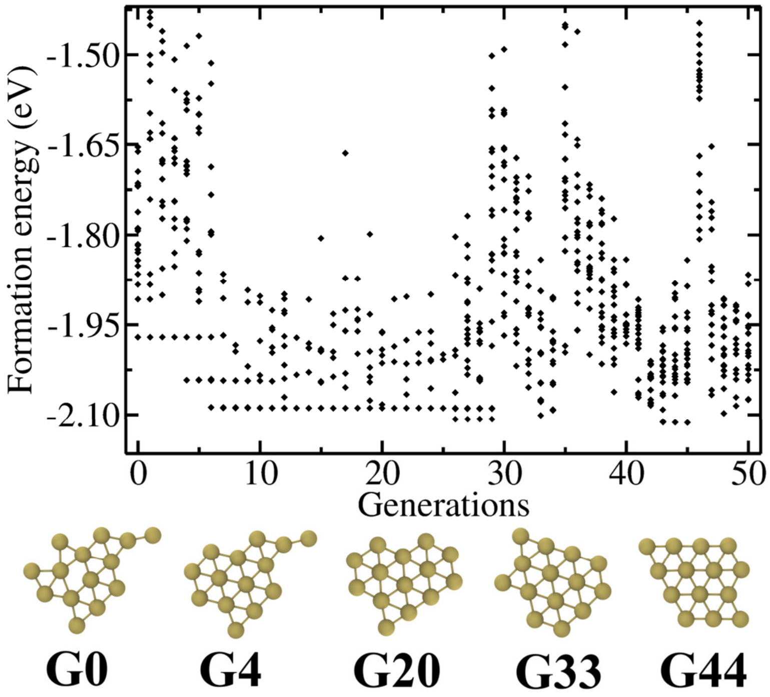

Figure S3: Per atom formation energies and structures of 2D Au_{14} clusters as a function of generation number through genetic algorithm optimization. The lowest energy clusters at selected generations (given by G#) are also presented in the bottom panel.

Structures of neutral clusters in xyz format

The Supplementary Dataset file Au_nanoclusters.doc contains the Cartesian coordinates of the clusters presented in Tables 1, 2 and 3 in the main text. The file may be opened as a text file to read the coordinates. The coordinates are presented in .xyz format. The first line denotes the number of atoms in the cluster; the second line is the name of the cluster i.e. the name given in Tables 1-3; following lines contain the elements symbol, x, y and z coordinates in Angstroms for each atom in the cluster. The structures are placed directly end-to-end in the file following the order in Tables 1, 2 and 3.

GA optimization of anionic Au_{13}

In Table S1 we present the anionic 13-atom gold clusters that have energies within $2k_BT$ of the identified minimum for this system. These structures are obtained as a result of 4 independent GA optimizations with Au atoms having freedom of motion in three dimensions. The DFT and GA calculations have similar setups as the ones for neutral clusters except the clusters have an excess electron.

Table S1: Au_{13}^- that are in $2nk_BT$ (n=13, T=300K) proximity of the predicted minimum energy structure for the anionic clusters. The ΔE is described as the per atom energy difference of the corresponding structure from the minimum which is given as Au_{13}^-(1). If a structure is same as one of the neutral clusters, the name of the neutral cluster (i.e. shown in Table 2) is given in brackets.

Au_{13}^-(1) [Au_{13}(1)]	Au_{13}^-(2) [Au_{13}(2)]	Au_{13}^-(3) [Au_{13}(3)]	Au_{13}^-(4) [Au_{13}(4)]
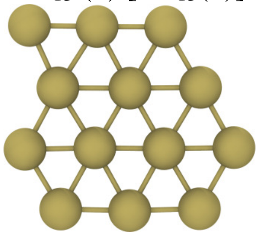	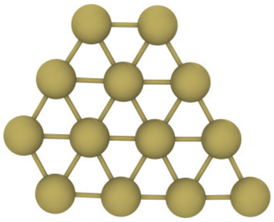	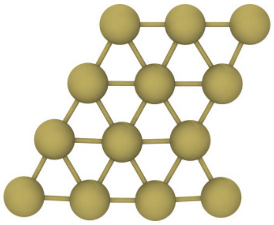	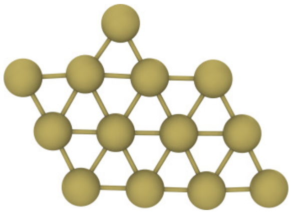
ΔE=0.000 eV	ΔE=0.003 eV	ΔE=0.014 eV	ΔE=0.019 eV
Au_{13}^-(5)	Au_{13}^-(6)	Au_{13}^-(7) [Au_{13}(6)]	Au_{13}^-(8)
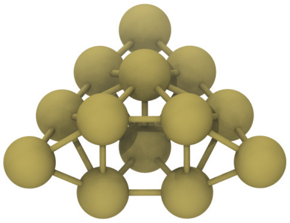	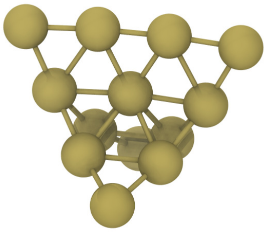	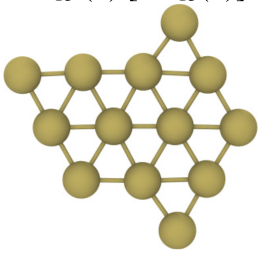	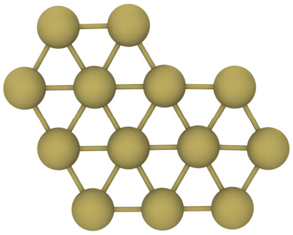
ΔE=0.021 eV	ΔE=0.024 eV	ΔE=0.025 eV	ΔE=0.028 eV
Au_{13}^-(9)	Au_{13}^-(10) [Au_{13}(7)]	Au_{13}^-(11)	Au_{13}^-(12)
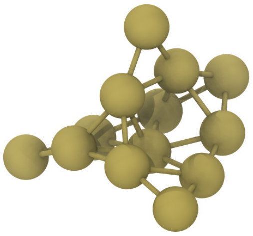	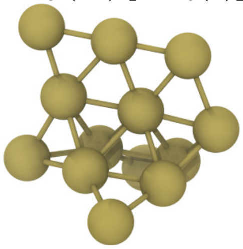	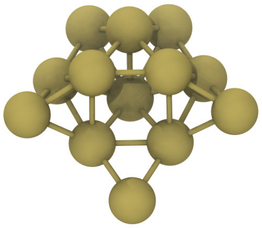	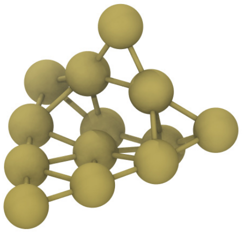
ΔE=0.029 eV	ΔE=0.031 eV	ΔE=0.032 eV	ΔE=0.033 eV
Au_{13}^-(13) [Au_{13}(8)]	Au_{13}^-(14)	Au_{13}^-(15) [Au_{13}(10)]	Au_{13}^-(16)

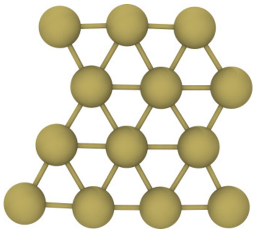
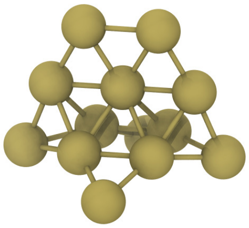
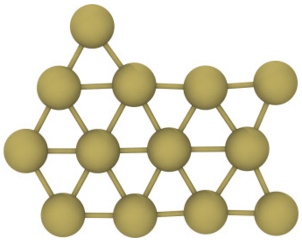
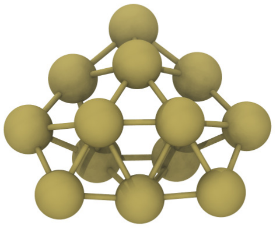

ΔE=0.033 eV | ΔE=0.034 eV | ΔE=0.035 eV | ΔE=0.035 eV

Au_{13}^{-}(17) [Au_{13}(11)] | Au_{13}^{-}(18) | Au_{13}^{-}(19) | Au_{13}^{-}(20) [Au_{13}(12)]

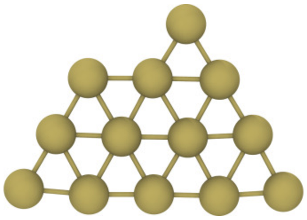
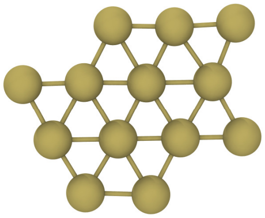
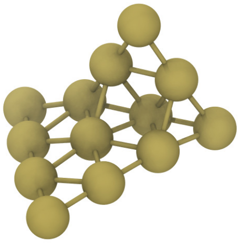
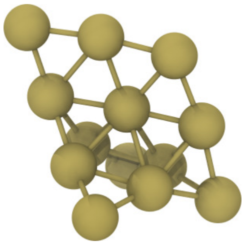

ΔE=0.036 eV | ΔE=0.038 eV | ΔE=0.041 eV | ΔE=0.042 eV

Au_{13}^{-}(21) | Au_{13}^{-}(22) | Au_{13}^{-}(23) | Au_{13}^{-}(24)

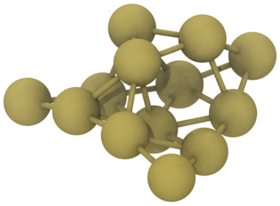
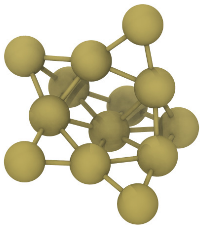
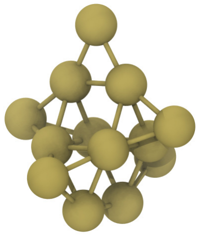
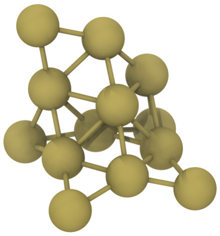

ΔE=0.042 eV | ΔE=0.043 eV | ΔE=0.043 eV | ΔE=0.043 eV

Au_{13}^{-}(25) | Au_{13}^{-}(26) | Au_{13}^{-}(27) | Au_{13}^{-}(28)

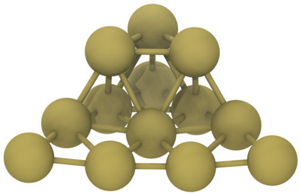
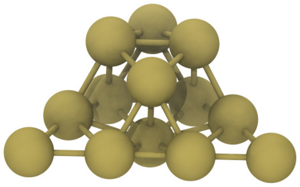
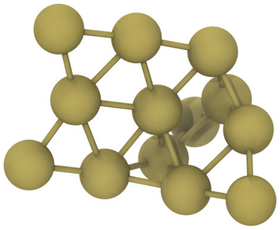
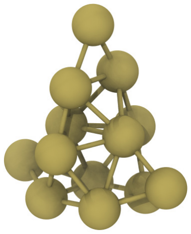

ΔE=0.045 eV | ΔE=0.046 eV | ΔE=0.048 eV | ΔE=0.048 eV

Au_{13}^{-}(29) | Au_{13}^{-}(30) [Au_{13}(13)] | Au_{13}^{-}(31) [Au_{13}(14)] | Au_{13}^{-}(32)

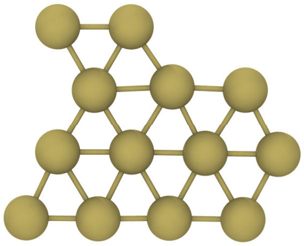
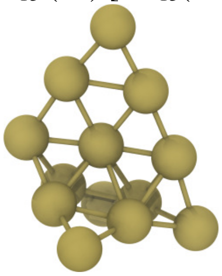
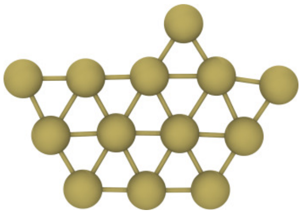
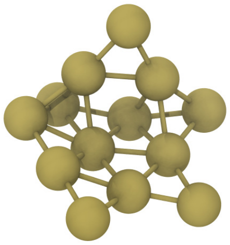

ΔE=0.049 eV ΔE=0.049 eV ΔE=0.049 eV ΔE=0.050 eV

Au_{13}^{-} (33)

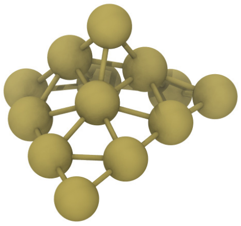

ΔE=0.050 eV

Au_{13}^{-} (34)

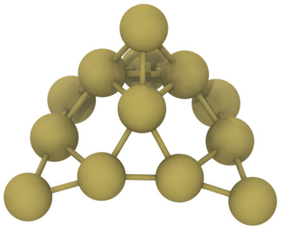

ΔE=0.051 eV

Au_{13}^{-} (35)

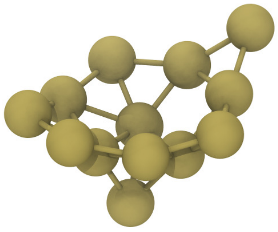

ΔE=0.051 eV

Au_{13}^{-} (36)

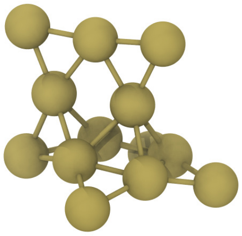

ΔE=0.051 eV

Structures of anionic clusters in xyz format

The Cartesian coordinates of the clusters in Table S1 are included in the Supplementary Dataset file Au_nanoclusters.doc as just after the neutral clusters. The format and the naming scheme are same as the ones for neutral clusters. The structures are placed directly end-to-end in the file with the order given in Table S1 following the structures given for neutral cluster in Tables 1, 2 and 3.

Hybridization in *s-d Orbitals*

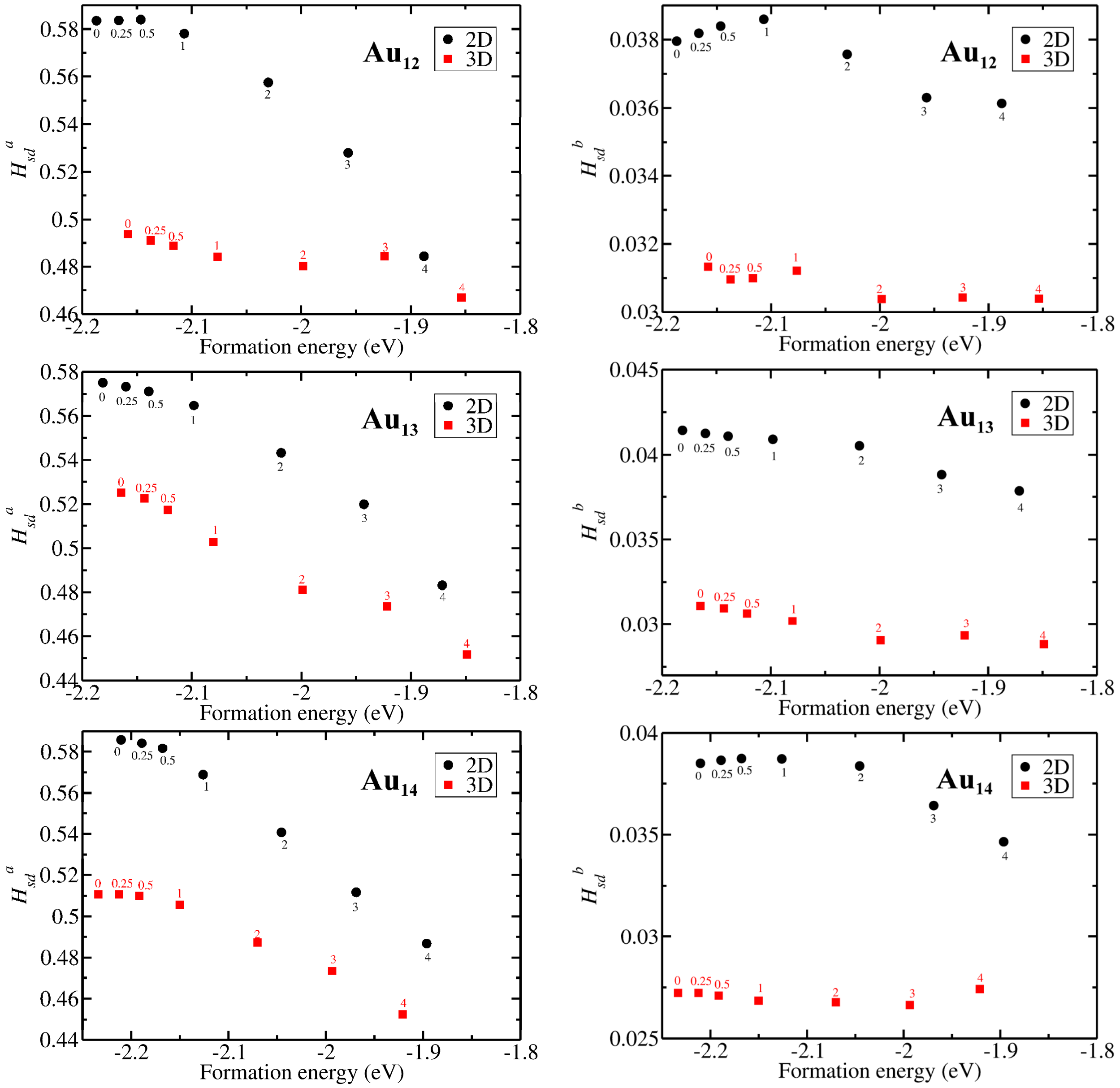

(a) **(b)**

Figure S4: The variation of *s*-*d* band hybridization index, H_{sd}, with respect to energy for different values of Hubbard correction term U. (a) H_{sd}^{a} is the common area under *s* and *d* shell decomposed electronic density of states while (b) H_{sd}^{b} is the product of *s* and *d* weights of local charges for different sized Au clusters in 2D and 3D constructions. The U values corresponding to each data point is indicated next to it.

Long Range Interactions

In Table S2, cohesive energies calculated from PBE+vdW are given for bulk gold. D2, D3, TS and DF vdW approximations are given with respect to different vdW interaction cutoff values from 3.8 to 21 Å. The cohesive energy does not change after ~15 Å appreciably.

Table S2: The cohesive energy of FCC gold from PBE calculations including the contribution of different flavors (D2, D3, TS and DF) of van der Waals interactions. The contribution of vdW is calculated for considering several interaction cutoffs. In parenthesis the percent contribution of the vdW to the cohesive energy is given.

vdW cutoff (Å)	Cohesive energy/atom (eV) with vdW				
	No vdW	D2	D3	TS	DF
21		-3.684 (18.9%)	-3.631 (17.7%)	-3.410 (12.4%)	
19		-3.682 (18.9%)	-3.630 (17.7%)	-3.409 (12.4%)	
17		-3.680 (18.8%)	-3.629 (17.7%)	-3.408 (12.4%)	
14.9		-3.676 (18.7%)	-3.626 (17.6%)	-3.405 (12.3%)	
3.8		-3.088 (3.3%)	-3.125 (4.4%)	-3.052 (2.1%)	
Not applicable	-2.987 (0%)				-3.550 (15.9%)

Details of DFT calculations

During the genetic algorithm optimization, we use an energy cutoff of 230 eV for DFT calculations. To test the adequacy of this cutoff energy, we randomly generate 1000 Au_{13} clusters and calculated the cohesive energies using 230 and 520 eV cutoff. Between these two sets, we find that %99.4 of all binary comparisons (i.e. between any two clusters within a set) in terms of energy order is same. We also investigate the changes in the order and the relative differences in energy for Au_{13} clusters around the identified minimum with respect changes in cutoff energy, see Figure S5. It is seen that the order and the relative differences in the formation energy for these low energy structures are virtually unchanged.

In cluster simulations, the size of the surrounding vacuum should be large enough to minimize the spurious periodic image interactions. The smallest cluster-cluster distances observed when the structure is constrained to 2 dimensions since these clusters have the largest diameter. We searched all our structures generated during GA optimization and the largest Au-Au separation in a cluster is found to be 21.5 Å for a 2D structure as expected. This leaves at least 8.5 Å between the atoms of the periodic image clusters since the simulation box has an xy plane with dimension 30×30 $Å^2$. In order to quantify the error introduced by the image interactions, we model the same cluster configuration in a 60×60×15 $Å^3$ cell. The energy difference between the calculations utilizing 30×30×15 $Å^3$ and 60×60×15 $Å^3$ simulation boxes is found to be 1.65 meV/atom. Note that such open structures have very high energies and around global minima, the clusters are much more compact. Thus distances between the image clusters are much larger in compact clusters. In this case the errors for the low energy structures are expected to be insignificant.

To be more rigorous in quantifying the effect of box size on our calculations, we also investigate energy differences between lowest energy 2D and 3D structures for Au_{12}^-, Au_{13}^- and Au_{14}^- within 30×30×30, 40×40×40 and 50×50×50 $Å^3$ simulation boxes. We relax the structures using the same criteria defined in the main text and calculate per atom formation energies according to Equation 1. As it can be seen in Table S3, the change in box size does not affect the energy difference between clusters and the largest discrepancy is about 1 meV/atom.

Table S3: Differences in per atom formation energies (E_f) between lowest energy 2D and 3D Au_{12}^-/Au_{13}^-/Au_{14}^- clusters in various simulation boxes.

	Au_{12}^- E_f^{2D}-E_f^{3D} (eV)	Au_{13}^- E_f^{2D}-E_f^{3D} (eV)	Au_{14}^- E_f^{2D}-E_f^{3D} (eV)
30×30×30 Å^3 box	-0.033	-0.021	0.012
40×40×40 Å^3 box	-0.033	-0.020	0.012
50×50×50 Å^3 box	-0.033	-0.021	0.012

After GA optimization, clusters within $3nk_BT$ of the identified minima are revaluated by fully relaxing their atomic coordinates. For these simulations, we use 30×30×30 Å^3 boxes. We also increase the planewave cutoff energy to 300 eV and consider spin polarization. The ionic relaxation is continued until the energy is converged within 10^{-4} eV/atom.

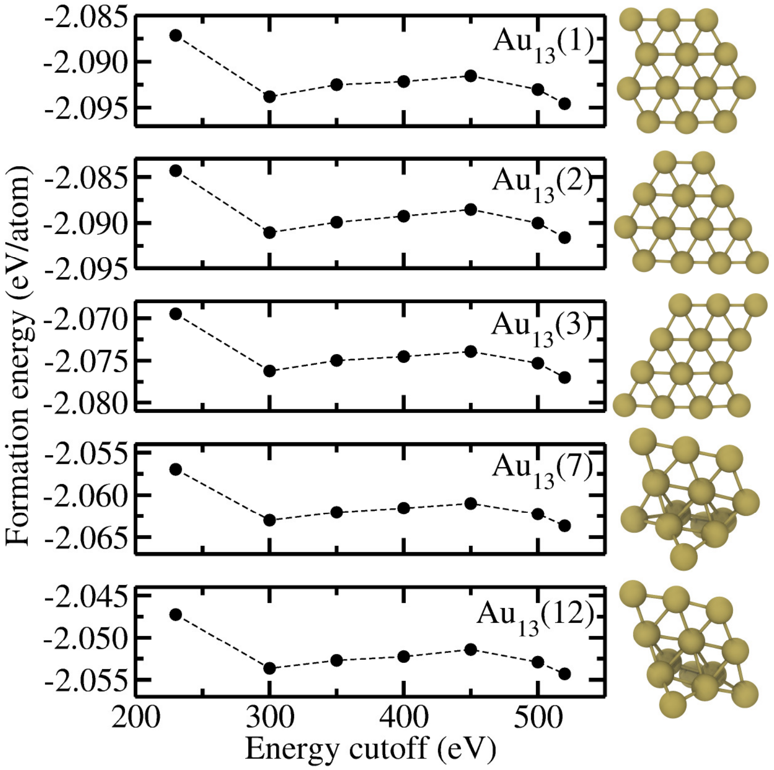

Figure S5: Per atom formation energies of low energy Au_{13} clusters as a function of planewave kinetic energy cutoff. The reference for the formation energy calculation is the isolated Au atom. The atomic structures corresponding to these clusters are given on the right side of each plot.
