# Supplementary material for: Unraveling the Planar-Globular Transition in Gold Nanoclusters through Evolutionary Search
Source: Sci Rep. 2016 Nov 28;6:34974. doi: 10.1038/srep34974 (PMC5124999; doi:10.1038/srep34974)
Supplement: Supplementary Dataset [file srep34974-s2.doc]

12

Au12(1)

Au 16.497139013771381144 18.462017401583317167 15.426895931497931258

Au 18.439446682160561153 18.011305723626318098 17.256157562520520798

Au 16.926784710509416243 15.914937706098559289 16.411880156563793065

Au 18.888412666379007732 15.519178440530357221 18.253385253397699017

Au 17.415866633665874730 13.398185948379548904 17.447059650252654706

Au 15.881190169632153797 11.350041788473737014 16.570544729624426594

Au 14.527887224670438471 18.796844375937414640 13.601708607853106869

Au 13.015686709644215924 16.698419230667109758 12.755178262152746527

Au 14.981790833185124612 16.305473367290087339 14.592968536335138907

Au 13.426164119224418769 14.199158893318223562 13.704688734146067475

Au 15.429646409191839496 13.840668669359519782 15.575702433777181000

Au 13.915261556965846879 11.745340339735836466 14.731325006878773110

12

Au12(2)

Au 16.203052229406445406 18.572348678105495168 15.960034590716265512

Au 18.425967736211461556 18.020962978324565285 17.388695352244262438

Au 16.982507890174520782 15.967535103847415812 16.341500860211525037

Au 19.247440378113566339 15.486112829666719648 17.817148706885753029

Au 17.854554249699301494 13.416385447238027950 16.792522880472624536

Au 16.292769880910128677 11.362307226448313813 15.659994208770191548

Au 12.507369984469105972 16.820465894718584110 13.413865148204489941

Au 14.747674973741057514 16.571252543912631694 14.886971063267301574

Au 18.437302633210197911 10.799566171978963069 17.043724981644668759

Au 13.330061850524817402 14.375997627373807930 13.835269470110389989

Au 15.557153365074821494 13.937628192850755937 15.302417638343047912

Au 14.079430593464286048 11.827233153534738364 14.215165198130073065

12

Au12(3)

Au 19.582387777176510468 19.957912344511903768 7.500000000000000000

Au 21.171791681448134170 17.781465251645020231 7.500000000000000000

Au 22.577153668221264127 15.532708905919497155 7.500000000000000000

Au 23.963188615361211475 13.251047142289211322 7.500000000000000000

Au 21.340209790063493500 13.022902786532117858 7.500000000000000000

Au 20.022207305729985194 10.710233833814271520 7.500000000000000000

Au 18.292863054725167160 17.674457100784444208 7.500000000000000000

Au 19.855665922098388876 15.436763755433428713 7.500000000000000000

Au 17.084790515131192734 15.297912632638565711 7.500000000000000000

Au 18.590914729822102913 13.052626279587446589 7.500000000000000000

Au 15.792518854332527667 12.935749858925172262 7.500000000000000000

Au 17.304506193890272669 10.700122258919073204 7.500000000000000000

12

Au12(4)

Au 16.248370659225177093 15.898326411067985831 13.945898591973701741

Au 17.867229611664459554 15.402504071344630177 16.022275869261296322

Au 18.250250282053318784 15.617513616431347501 18.743845886423205371

Au 17.084408372281821187 13.357417961958816477 17.855115067301095877

Au 16.479889252904282415 13.327337643341797602 14.879130944836257200

Au 15.805637013417166870 11.320609468552401466 16.772186114486522968

Au 13.850544118064677335 16.964676710077295496 13.099971341429204941

Au 14.031777073829799107 16.324183310123991930 15.775059623639156925

Au 15.650275711758785846 15.827306292380903585 17.851706839616930722

Au 13.862584440484598147 14.344134028036874540 13.725014586987271059

Au 14.319888904328447410 13.741641858833906653 16.662142770540580017

Au 14.073171658986931121 11.849759082849887548 14.555882337504710833

12

Au12(5)

Au 19.450544817212708892 19.929815573246202121 7.500000000000000000

Au 20.988709856903639661 17.777598607710057621 7.500000000000000000

Au 22.486547181488106162 15.544918622708808087 7.500000000000000000

Au 19.700872679889119610 15.366672836345502517 7.500000000000000000

Au 21.315297021684418866 13.149427824490874883 7.500000000000000000

Au 20.063560091518475303 10.799711877395063553 7.500000000000000000

Au 16.622041849024999038 19.820939138948173053 7.500000000000000000

Au 18.158873536238001378 17.566130665622086582 7.500000000000000000

Au 15.407689586229400547 17.455319702605432752 7.500000000000000000

Au 16.919562988795505021 15.086818602374552967 7.500000000000000000

Au 14.225176830023807284 15.128234822989849562 7.500000000000000000

Au 18.510496252992126642 12.946643448563746048 7.500000000000000000

12

Au12(6)

Au 20.691829899018500782 17.973336385863117215 7.500000000000000000

Au 22.163464894808040384 15.794322494976025695 7.500000000000000000

Au 19.471167091892752410 15.481915984667875108 7.500000000000000000

Au 23.884846717738991373 13.823456277260147473 7.500000000000000000

Au 21.327739492278229960 13.099536215630322999 7.500000000000000000

Au 20.010552855812743900 10.772101420320598919 7.500000000000000000

Au 16.254869292595842012 19.683900446261048955 7.500000000000000000

Au 17.980431036019162150 17.693189667008066834 7.500000000000000000

Au 15.229794715939760863 17.254864746087726246 7.500000000000000000

Au 16.809439867760655574 14.960745617272683816 7.500000000000000000

Au 14.119304284987151732 14.905836932808053774 7.500000000000000000

Au 18.563007190148237413 12.959884361844624223 7.500000000000000000

12

Au12(7)

Au 20.690593429652356150 17.920959782926814796 7.500000000000000000

Au 22.329634558188903526 15.772048938515094818 7.500000000000000000

Au 19.634907791445851188 15.415625565372220507 7.500000000000000000

Au 23.973726964059572708 13.710642457278698458 7.500000000000000000

Au 21.334069538648179787 13.210647093160105214 7.500000000000000000

Au 20.312798010952111127 10.805306195223428389 7.500000000000000000

Au 19.136205484634757568 20.051323306003776992 7.500000000000000000

Au 17.957850929752034119 17.636979835100472513 7.500000000000000000

Au 15.367879447728661546 17.290735303547108970 7.500000000000000000

Au 16.891023334457219818 15.079934484644690329 7.500000000000000000

Au 18.584739102995250448 12.859204044017166879 7.500000000000000000

Au 15.951132425485109678 12.606867480210333099 7.500000000000000000

12

Au12(8)

Au 17.839006217901765439 15.244012317671982615 15.694471518393550369

Au 19.465297189513119491 14.806251503762691613 17.773677681411300000

Au 15.899779676380966009 15.766430102496780563 13.956355407952930392

Au 17.137643546481225343 13.571821023219539981 17.900622450728107538

Au 15.873629556914593763 13.376555658489710510 15.336232379200339437

Au 15.109824851211678620 11.822771246299046410 17.521260770163944898

Au 12.696286166135241302 16.366906722540608854 17.920596897789238255

Au 13.520498509365998174 15.901261816490141143 15.464646415847248306

Au 13.455024032356762476 16.332461506261889639 12.777787020669336471

Au 14.510440793817473093 14.468518513358134214 17.638700988352240984

Au 13.725383242125936079 13.788260883272467794 13.609505699061692496

Au 13.993481140794861162 11.460931462137669001 14.870118317430586430

12

Au12(9)

Au 16.083087406318096413 18.015281208584148942 15.230200302560662706

Au 18.271737150310315201 17.944465581095059292 16.833765791368449527

Au 16.520982798114747681 15.916747844962022995 16.995160635107779967

Au 19.117911645501273199 15.490086849135698444 17.768454649333051520

Au 17.506941769247806207 13.375068237655208137 17.400604135279319706

Au 16.040131062572712040 11.369766928910165049 16.313352821657783664

Au 15.322898125209441389 19.249600961197160132 13.000740626768015673

Au 14.091944479150919634 16.944918482390939118 13.330326637242951193

Au 14.035625068252565839 16.302071120338858634 15.984615320026632546

Au 13.334719476711224928 14.450897986554867458 13.996422425532074385

Au 15.029328052692358497 13.809634724224981284 16.105796296123855882

Au 14.041181740918583287 11.856625011950612247 14.470053497999352032

13

Au13(1)

Au 18.342703168298825744 17.122768394358743649 7.500000000000000000

Au 16.503885149045196101 19.009044635794730027 7.500000000000000000

Au 20.227177257832597235 15.235647410358749809 7.500000000000000000

Au 17.557112012914469545 14.520511706619206649 7.500000000000000000

Au 19.530140665671098077 12.605282365249252052 7.500000000000000000

Au 18.800157104357232640 10.026268928765228239 7.500000000000000000

Au 15.578785758228310243 16.502628594618975910 7.500000000000000000

Au 12.984541446089847838 15.706348762669414043 7.500000000000000000

Au 14.951197978996194848 13.831956771325833344 7.500000000000000000

Au 12.328906371517209806 13.075474997311239633 7.500000000000000000

Au 16.839676036541863624 11.913194146550763364 7.500000000000000000

Au 14.192427466576040374 11.142652577382131440 7.500000000000000000

Au 16.168033137931264065 9.280959789995787190 7.500000000000000000

13

Au13(2)

Au 14.189427529496967395 14.255496899600522909 7.500000000000000000

Au 16.571345007689039619 12.980237444348928122 7.500000000000000000

Au 18.973013559049093146 11.710846551852768016 7.500000000000000000

Au 14.240619777079896124 11.496200755376595026 7.500000000000000000

Au 16.658666388298957628 10.261959098385791123 7.500000000000000000

Au 11.898386809654970975 12.769093296574256513 7.500000000000000000

Au 16.489986641812006241 15.671213593632270289 7.500000000000000000

Au 14.072571335309547180 17.013209063378617714 7.500000000000000000

Au 9.598382424197769325 14.116893355648857167 7.500000000000000000

Au 18.958170390573442887 14.399510425359510535 7.500000000000000000

Au 11.812848871481381963 15.599960304622351615 7.500000000000000000

Au 16.413554120711939532 18.388026797426888947 7.500000000000000000

Au 18.812224644645240801 17.083754913793729457 7.500000000000000000

13

Au13(3)

Au 17.152114242006042844 16.944827556968842686 7.500000000000000000

Au 19.664915566596246066 17.727502298631069522 7.500000000000000000

Au 19.306289438806565784 15.085213556585769012 7.500000000000000000

Au 18.746143477464968896 12.498266357248754233 7.500000000000000000

Au 16.125251373988923831 11.610127852181644670 7.500000000000000000

Au 20.631866072048218541 10.656260071680861756 7.500000000000000000

Au 18.122317427423592306 9.742870012911005517 7.500000000000000000

Au 16.687529354864285125 14.271177822734701479 7.500000000000000000

Au 14.618866185260355195 16.159193008846411743 7.500000000000000000

Au 14.083886529404409771 13.490327540168237874 7.500000000000000000

Au 12.979109358420222620 8.308371467353218520 7.500000000000000000

Au 13.470333902448510699 10.902850251709894636 7.500000000000000000

Au 15.572435274267670380 8.974719023979300658 7.500000000000000000

13

Au13(4)

Au 18.536233742606075481 17.948443951908515714 15.054978839346023989

Au 16.125865774427335708 17.763111781178007931 13.756667488320101000

Au 19.740120427462777286 15.670644170185942912 15.807943685783673260

Au 17.371814795164645773 15.483784410777163387 14.549549939569301316

Au 21.010203246800493559 13.506796815152840807 16.587314035686546276

Au 18.673036771138601608 13.107786882940217765 15.362863195755981138

Au 17.470597309944896125 10.793454685011438343 14.834666962817726343

Au 13.810029977302818338 17.601237339346003097 12.495791180041626589

Au 11.480362729770781982 17.259854372223415453 11.224939139168384372

Au 14.995347632970389640 15.261644542856970475 13.278044833602152153

Au 12.620463505875816779 15.023612168549165702 11.985909568368342093

Au 13.842257893168810057 12.798543099939701406 12.775691684297463269

Au 16.233541136367051649 12.994565884930441513 14.060759502242309082

13

Au13(5)

Au 16.338087014420725751 18.017606828409473252 7.500000000000000000

Au 18.311456040599900064 16.170505335724442375 7.500000000000000000

Au 15.645230715612010286 15.422787110300214763 7.500000000000000000

Au 17.626100321925292747 13.559630034409355659 7.500000000000000000

Au 19.467985707862744960 11.625433498369121210 7.500000000000000000

Au 16.944522678926745130 10.871102004156741216 7.500000000000000000

Au 16.154754790184821189 8.336901146139302554 7.500000000000000000

Au 13.706730615111549554 17.402144484480363218 7.500000000000000000

Au 12.982909405514144296 14.824914149364287752 7.500000000000000000

Au 10.472975431079847297 13.958473709520992756 7.500000000000000000

Au 14.965169049417557545 12.821460566947179061 7.500000000000000000

Au 12.352589765947111999 12.090516583644077997 7.500000000000000000

Au 14.245329848397638273 10.196840425534263730 7.500000000000000000

13

Au13(6)

Au 18.221831437057524283 19.115045207833503582 15.045574647651152489

Au 19.061879214684708472 16.702094370452261529 15.588463086952945247

Au 17.564491060159362235 14.593758348721713958 14.822793642488109711

Au 19.992594038132963874 14.240636969656508271 16.178091374750710685

Au 18.533802297008151072 12.136531321091212021 15.430869176939495446

Au 17.169988033856657950 10.011364270306373214 14.744486454440105305

Au 14.290761794362571990 17.396677683277768978 12.935372662743326089

Au 11.956906208930819346 17.554347327214554042 11.632634651924025349

Au 16.605897358933319197 17.137294347555481977 14.216788089224207781

Au 12.829202830040120631 15.118367572248471831 12.183551605289583364

Au 15.216950432592003750 14.882012276535183659 13.512271280494250192

Au 13.728461103228804419 12.687689922281483845 12.746019233471930221

Au 16.072141752012694127 12.353681445825257512 14.056703086630088606

13

Au13(7)

Au 19.604420313582782143 14.777604312653060958 14.662681227048167898

Au 17.504085115277774776 15.517517422098377367 16.456577223743103389

Au 17.174859164699174841 13.560196687596006271 14.528619895129356365

Au 15.956116602070773425 13.345617013827565600 17.069490519878950607

Au 15.048056861411403418 15.260260901030317626 15.271185420194761662

Au 13.115253177957930220 15.483423814527336404 13.472921712024827912

Au 19.164216146653615169 12.840751288873379465 12.844173515060264279

Au 18.724422287799395548 10.930022576478624075 11.060200566375048226

Au 16.684227418406614163 11.523456286506860380 12.643468348789170363

Au 15.402278890667538747 11.316428341075885911 15.192915877625322807

Au 15.310669319198726868 10.884343133382371249 17.804123933547678860

Au 14.502113213620535959 13.227705693516517371 13.391801793671881171

Au 14.177480804653551871 10.555117667433176365 12.853922279911031978

13

Au13(8)

Au 14.970495427001198507 14.243791310666301442 7.500000000000000000

Au 12.448966319707260197 15.509653655513677251 7.500000000000000000

Au 15.157175462208126504 11.510014430544833530 7.500000000000000000

Au 12.723397517641544141 12.774429044713860648 7.500000000000000000

Au 12.438214036308746557 18.140161680717884707 7.500000000000000000

Au 17.401689228504718443 12.947549122277663969 7.500000000000000000

Au 10.495423931469611389 11.252961820663280434 7.500000000000000000

Au 14.847386765454341884 16.973435375963596528 7.500000000000000000

Au 12.825807231689690724 9.976080579079976474 7.500000000000000000

Au 19.547778392962158023 14.449968863512490103 7.500000000000000000

Au 10.254289829288341096 13.994941434683443759 7.500000000000000000

Au 15.228158872668926094 8.856724182731895212 7.500000000000000000

Au 17.202659185094798744 15.780439898931451381 7.500000000000000000

13

Au13(9)

Au 18.305585357952629977 19.151417531853894616 7.500000000000000000

Au 21.887832543243327166 15.949913071052112201 7.500000000000000000

Au 19.336418242808587564 16.714323822173589917 7.500000000000000000

Au 17.432775366193542510 14.591522775821124114 7.500000000000000000

Au 20.069529435485200963 14.016196318928734854 7.500000000000000000

Au 18.268959355126607846 11.988037055972583289 7.500000000000000000

Au 16.562780249729598836 17.159829030404630146 7.500000000000000000

Au 14.746020246264206932 15.210564225647877024 7.500000000000000000

Au 15.568065976067259726 12.605630764144706291 7.500000000000000000

Au 12.871232973908627528 13.196193673576134486 7.500000000000000000

Au 13.667553743198595839 10.656521839065460355 7.500000000000000000

Au 16.447083393457660350 10.017387182373491683 7.500000000000000000

Au 14.508591081564826908 8.164807098985496836 7.500000000000000000

13

Au13(10)

Au 15.221901769973731788 15.292807525740311547 7.500000000000000000

Au 17.720114363221263432 16.795680618296607634 7.500000000000000000

Au 19.990699272537447939 18.194251010853385253 7.500000000000000000

Au 22.442321342939060003 16.894520984790581508 7.500000000000000000

Au 17.567475345765096506 19.569360531485081367 7.500000000000000000

Au 17.722334577102294872 14.112691838820012435 7.500000000000000000

Au 19.904493185367481800 20.916736665584139132 7.500000000000000000

Au 22.538733259946518217 14.284479564727002199 7.500000000000000000

Au 20.078789609823761708 15.371858422600841010 7.500000000000000000

Au 15.355712703534306840 18.113812256498043496 7.500000000000000000

Au 22.315850254690346333 19.557249398244334060 7.500000000000000000

Au 13.054020891465810905 16.827483089961155827 7.500000000000000000

Au 15.557868023633217192 12.629881592398472634 7.500000000000000000

13

Au13(11)

Au 15.805678307950879358 19.808379654001971204 7.500000000000000000

Au 17.165130966070933027 17.498191675280665436 7.500000000000000000

Au 18.505489934281847297 15.205487594610305635 7.500000000000000000

Au 15.819720175131559259 15.119421969336904965 7.500000000000000000

Au 19.734525529316332637 12.835137309436868591 7.500000000000000000

Au 17.087639281901001453 12.698573385998455976 7.500000000000000000

Au 14.397582576123330256 17.586950038371263361 7.500000000000000000

Au 11.734035345405327888 17.626155483802563140 7.500000000000000000

Au 10.390229717689130950 15.326938216437222096 7.500000000000000000

Au 13.088461429499179900 15.194187893974044457 7.500000000000000000

Au 9.031267995936188342 13.081558600481562493 7.500000000000000000

Au 11.719232193824565158 12.817702432519984868 7.500000000000000000

Au 14.396678554869492217 12.790177928747946012 7.500000000000000000

13

Au13(12)

Au 18.675097125987463187 14.532395117988368582 15.142800931117722740

Au 17.375281343896908481 16.020092181409122389 17.051874180568749040

Au 16.979760576459653976 12.847362471593907429 13.802526089169008472

Au 16.426226211878763195 13.509336426015671861 16.697249331696454533

Au 15.956841233228345800 15.517665065803406321 14.798968961530647448

Au 14.354648704867592812 13.711910206294227521 13.329244346729240078

Au 14.034643177789000390 16.338289821104435617 13.187789226592856906

Au 19.581751270034246204 12.903976839755795680 13.165380155061935596

Au 17.970570629934293549 11.225668787060055109 11.789812222716701484

Au 16.136583657511557988 9.679191833074838769 10.618101709362200324

Au 14.815921686383388689 11.737114454499558036 15.194565757146897056

Au 15.168044370147766742 11.472132763478398942 17.807955860746641719

Au 15.151507927880574655 11.217029520922128683 12.525809668560791366

13

Au13(13)

Au 17.875133894191947093 15.428979098627360500 16.550806176972102435

Au 20.041976162209987677 14.395617959029067379 15.309489990085092259

Au 15.828141078835296085 13.567619509568936920 16.877649012170984122

Au 17.505410286963059008 13.516569414644921565 14.616887907051351547

Au 19.517552656412860301 12.342661257374571093 13.287944272885452435

Au 17.004018524946154400 11.594671144223987369 12.778914904258840579

Au 21.960926104610884124 13.192549672882856626 13.942312548335534572

Au 14.193415293878231864 16.112200145744889568 12.784669931522046227

Au 15.577966221576598826 15.525128653920809541 14.972858729947301271

Au 14.896679056242362549 13.579035802780115461 13.047708094392115541

Au 15.134818504915223514 11.590118262644624636 14.995494987062237158

Au 14.812811679654082297 11.222242453401722884 17.591583970343929622

Au 14.340739623563035110 10.990557689155682652 12.474947527973487382

13

Au13(14)

Au 15.911597371767502551 19.926366497680895407 7.500000000000000000

Au 17.117341911095781626 17.594493645586499753 7.500000000000000000

Au 18.439205818374652779 15.263696683151902533 7.500000000000000000

Au 15.722872366251936072 15.256677409666135503 7.500000000000000000

Au 14.416399979260294373 12.906462147778036353 7.500000000000000000

Au 17.170609588690417979 12.868576157776395519 7.500000000000000000

Au 15.846703152761449473 10.529878184396954666 7.500000000000000000

Au 14.315462819776639591 17.779577642676951399 7.500000000000000000

Au 11.703500197035928920 17.616139111752669777 7.500000000000000000

Au 12.976255608628900262 15.283352326271282706 7.500000000000000000

Au 11.755378428366459076 12.872748165766283890 7.500000000000000000

Au 10.523221879552824731 10.533735201439164086 7.500000000000000000

Au 13.192485140437982594 10.422914351056670412 7.500000000000000000

13

Au13(15)

Au 16.447820496989418615 15.607242591571424128 14.342860426951821395

Au 17.648701419335875329 15.278628199568732526 16.770594083511287664

Au 19.176525118588834573 15.163958095510141177 14.460897983034914915

Au 16.146031843868851041 12.856469945756979101 17.349772328104776875

Au 17.550368145485204963 13.010874304272505242 15.041948172047979071

Au 15.244308814037738742 11.559925807412909649 15.198777494162152735

Au 16.943293673009822697 14.581848435106287809 19.225644989517284955

Au 14.340640738008834987 16.097566712995199367 12.831352866951855063

Au 14.893418589662015705 13.554064669738245641 13.229516572168456889

Au 19.187839391914803144 12.981804703520756661 12.863659496566500096

Au 16.758459733172649919 11.502407531478290537 12.699054300730868050

Au 14.115655391476181535 10.992463861233552791 12.744315690510566341

Au 18.895139125449489370 10.838757679834811753 11.304895528741282007

13

Au13(16)

Au 15.943862349808640033 19.780119204863527926 7.500000000000000000

Au 17.205839304238519816 17.499059428369879043 7.500000000000000000

Au 18.615744175650878134 15.250608369971082823 7.500000000000000000

Au 15.817577717612209653 15.167099975101955067 7.500000000000000000

Au 17.298865160666029084 12.921410311596817522 7.500000000000000000

Au 15.975745180000494372 10.570744437238870361 7.500000000000000000

Au 14.389758574714017314 17.584281340330068844 7.500000000000000000

Au 11.689167455810999030 17.515061165843626156 7.500000000000000000

Au 10.406419519121227424 15.142222505622338957 7.500000000000000000

Au 13.127065255622811790 15.168258037450108588 7.500000000000000000

Au 18.679670373841819497 10.653485736936566752 7.500000000000000000

Au 11.729329024357252109 12.806069405673831341 7.500000000000000000

Au 14.430074960554964747 12.749123267001081672 7.500000000000000000

13

Au13(17)

Au 19.939763260163410052 14.556492802044317614 14.913447980195561726

Au 17.801473578542690746 15.468208329465364059 16.427010369346042751

Au 15.924944021232816382 13.548853101604461102 16.902403832553151375

Au 15.415379044143978859 11.641170383190431181 14.876847512440557253

Au 15.425113796864394544 15.576499287226456048 15.093139344624406206

Au 14.810704610945061077 11.206601080294996464 17.425988873809814095

Au 19.578039748941638010 12.731616891087758958 12.956435393289607916

Au 18.998412827342722409 10.934974206470521096 11.097313103163719106

Au 17.439070081769180831 13.591117037930970923 14.419294362020398381

Au 16.787472865552199863 11.726365947544524104 12.505468881157101535

Au 16.591674009508246002 9.932162090059028614 10.535962760011022610

Au 14.927460168789989936 13.653196391901392559 13.077615419466898317

Au 13.616957617203672370 15.960904971179141754 13.199274477921552062

13

Au13(18)

Au 19.234572390134825781 17.629799080069993522 12.980229741585260328

Au 19.210704344748435801 15.647897747826640114 14.674191182249495924

Au 16.804858402809571771 14.766586066304915548 15.783378435389355587

Au 19.311760818911778870 14.396812791632321193 17.045172721717349873

Au 18.594530026119542754 12.762655185496967647 15.057986945912976040

Au 17.790074271080339230 10.585855468987256600 13.846049837768349633

Au 14.343117959427367225 17.318780929292849180 12.752661388669826792

Au 12.005013772104087266 17.470073830093152623 11.435781750653228883

Au 16.796857550284489236 16.779843379604756137 13.702031711079365905

Au 14.801842395016844733 14.943144682978482862 14.000194720917226832

Au 12.648434353321826862 15.071341074437089702 12.334414582592126308

Au 13.361688497595471503 12.698273414196837194 13.315705619607246746

Au 15.947017753445889454 12.493272709078572902 14.195551898859196882

13

Au13(19)

Au 18.997824219957575309 20.588859477494427352 7.500000000000000000

Au 20.836256131472442377 18.587513656210983726 7.500000000000000000

Au 18.173232040811793553 18.008922996782096959 7.500000000000000000

Au 20.067487650774577190 16.029870005917477016 7.500000000000000000

Au 17.307615295973800329 15.421736382956192202 7.500000000000000000

Au 21.710624244438438524 13.962361461363483883 7.500000000000000000

Au 19.114196654006207154 13.393630880033411401 7.500000000000000000

Au 16.329851153139298248 20.067450064025781131 7.500000000000000000

Au 15.498689557883407630 17.517154160933479545 7.500000000000000000

Au 14.618798851985415510 14.995745702320222748 7.500000000000000000

Au 16.514961248307070463 12.841926497946284158 7.500000000000000000

Au 13.698672197522935079 12.521495838141648349 7.500000000000000000

Au 15.348465573726782551 10.447387610874264041 7.500000000000000000

13

Au13(20)

Au 19.963875200365123419 14.555732870067835449 14.905961272112767446

Au 17.768580892771094426 15.578332482944016846 16.303087224729413407

Au 17.425385632543083148 13.558402466566095157 14.550280679384712812

Au 15.819027559570940866 13.687652095888230619 16.861966363892040732

Au 15.355084532979304868 15.512717791027055725 14.809497526887467345

Au 15.346842070316261797 11.471540308636877015 15.204528090622632774

Au 14.696018973486326686 11.471037403446651126 17.779412419274194690

Au 19.551385072890443695 12.566210074342146541 13.120716497216983853

Au 18.983008506244381408 10.651103734466229156 11.282832307121726245

Au 17.034307912945095609 11.417846029633839322 12.984843207011330435

Au 16.437018415354685885 9.578917835079996834 11.118817584793166731

Au 14.818368442260096529 13.389039873874800435 13.265520347207631957

Au 14.428336223273376504 10.699670933026265374 12.653333626745775220

13

Au13(21)

Au 20.063077032580277859 14.660300533104425824 14.616116793408131969

Au 17.989625547177858778 15.932338772480186151 15.858001509107172211

Au 17.506702189994470586 13.673890771977617931 14.400910294828930702

Au 15.450830365855829029 15.126838770374542165 15.627021918343880102

Au 13.090888916454550994 14.686655429288958175 14.364394172227544999

Au 12.896131611685536456 14.063278141081209327 11.774552102932666742

Au 19.713545626831251667 12.501362363347496398 13.120567254508539889

Au 19.268377411737617422 10.388035982469871144 11.604966585377448851

Au 17.125610515304295234 11.445245553572252817 12.799969621795764141

Au 15.717468591131009248 11.612282988659480054 15.162070098051572131

Au 15.062893372046849194 13.231824957035648538 13.071468918538121429

Au 14.330415616029418047 10.641482703972398483 17.202245786832655483

Au 13.806456369171071330 13.151540011635987071 16.497294082047140762

14

Au14(1)

Au 14.041857300670661957 13.578520956689100530 15.064948341065232285

Au 13.859150199130034764 16.291514116327796557 14.454377691188092570

Au 11.629384851273641743 14.660423311992843765 14.049778130608112647

Au 12.642583235783135365 12.048603475470164526 13.314126428797971258

Au 13.977060481685439797 14.431368960349463038 12.368346282077517628

Au 12.512874944470770089 12.965543598583831653 10.557560238950621567

Au 12.782702470538620076 15.635295185343112578 10.279824347842390964

Au 12.454044608005688133 17.365830329980237678 12.433083460302787771

Au 11.213237216754798453 10.630818838767687851 11.501319316764803347

Au 10.133975093352177055 15.852112625402000390 12.152602896219516282

Au 10.183202332921442945 13.160346693882081226 12.252368704493065366

Au 10.843255806901902716 17.606379327287669412 10.131065760128519671

Au 10.193019549541292079 14.934181186113747231 9.523970592202061880

Au 9.979246968970592491 12.258220997809923247 9.609117237358919539

14

Au14(2)

Au 13.413524432988383595 16.276895761195369516 13.533779056704799970

Au 14.133829551067295327 14.046957533847107769 11.994520401600087922

Au 12.747172397771805663 15.864930423272959104 10.533004263667615774

Au 11.744704566585790317 14.073202168505865473 14.020092673889699597

Au 10.571959607590404318 15.921103861328402829 12.407804355240706684

Au 10.458173600435907602 17.454918392855830689 10.029059748584051803

Au 10.116326810768768496 14.804261181756070087 9.684353072439938259

Au 14.421586669752443299 13.979477335277916694 14.756163460953576561

Au 13.226769879581789624 11.818280906297266242 13.380856293866475681

Au 12.404485175394261987 13.172632558200758623 10.148830093484672688

Au 9.899082302947329381 12.070440980599572711 9.523048714638695955

Au 11.530012596829548599 10.908918884309880326 11.494961618255450375

Au 10.124211826793258595 13.243074164806049708 12.060438188814357474

Au 12.217963913494022776 18.175302534747743266 12.054702904858720203

14

Au14(3)

Au 15.735740924173862609 20.038934216788543807 7.500000000000000000

Au 18.342160605652260585 20.682320018668431061 7.500000000000000000

Au 20.156372653367906622 18.677559586216869292 7.500000000000000000

Au 17.552884598896206114 18.000421159977857855 7.500000000000000000

Au 19.497264677716053427 16.052661261396803383 7.500000000000000000

Au 16.785250924660235938 15.405696746612386860 7.500000000000000000

Au 21.228889660665892336 14.026545681447894154 7.500000000000000000

Au 18.659097528052924986 13.402785974641599154 7.500000000000000000

Au 16.044328386134573350 12.785418426885781784 7.500000000000000000

Au 14.104361535448965981 14.712225650098091023 7.500000000000000000

Au 13.140844074641329087 19.412008600653365420 7.500000000000000000

Au 10.606564640018993018 18.710212539024556833 7.500000000000000000

Au 14.920846239866358829 17.366364201882639406 7.500000000000000000

Au 12.296410428704644957 16.637975847704439047 7.500000000000000000

14

Au14(4)

Au 17.539071181195446059 15.326655419472208663 7.500000000000000000

Au 14.877106656389891270 14.713258710379932381 7.500000000000000000

Au 19.309027320065979438 13.352386390795121685 7.500000000000000000

Au 16.784700209577430030 12.601992524884842339 7.500000000000000000

Au 14.127770879567936646 12.086055446220687060 7.500000000000000000

Au 15.810450967005941436 17.319144753692260252 7.500000000000000000

Au 15.956280491007206024 10.013656266914868098 7.500000000000000000

Au 13.373541405264884219 9.487961750963739505 7.500000000000000000

Au 13.105174431929095036 16.768385935770005801 7.500000000000000000

Au 10.534201197217290868 16.095927827530069010 7.500000000000000000

Au 12.272242468522714276 14.033176239709479916 7.500000000000000000

Au 9.592863783754628670 13.540127846610380047 7.500000000000000000

Au 11.327347323091625952 11.459216698249349520 7.500000000000000000

Au 10.846188685409979158 8.825354784807139197 7.500000000000000000

14

Au14(5)

Au 17.528977162577273674 15.466997940640766274 7.500000000000000000

Au 14.843613894450392365 14.746142374028448074 7.500000000000000000

Au 19.348188368124684899 13.421093244288927693 7.500000000000000000

Au 16.714720487850776465 12.799014090483744610 7.500000000000000000

Au 14.085550395949923086 12.182159387259000383 7.500000000000000000

Au 18.599220385397362776 10.846047586986896860 7.500000000000000000

Au 15.958747897664547821 10.121452676919812319 7.500000000000000000

Au 12.968929636068059708 16.805151573320937786 7.500000000000000000

Au 10.328240993945895454 16.079910934252183097 7.500000000000000000

Au 12.213772319586556847 14.128220396457930264 7.500000000000000000

Au 9.579788737740823734 13.504975212455418543 7.500000000000000000

Au 11.399875193232402282 11.460977906103954282 7.500000000000000000

Au 13.325761851931128277 9.582001146202902220 7.500000000000000000

Au 15.602039398480231469 17.346439699598342088 7.500000000000000000

14

Au14(6)

Au 12.264629743783995508 18.282699564418983584 10.854669562597234034

Au 12.805766967422124836 17.185726645779400457 13.244437083575924419

Au 12.551770157437832864 15.843755747445264603 15.597829400567590596

Au 13.157746201786199691 15.706513084871934538 10.886551353940582487

Au 13.190255482700155909 14.374349040136490174 13.394639629649605084

Au 12.420237684237779519 13.097430294888011915 11.059804343173063046

Au 12.800330480988350246 11.710122208353894280 13.429622466599516528

Au 10.701324230563585616 17.392605923220525455 8.737955933109704532

Au 10.205131351817518848 16.414684564813494916 11.254480174073252741

Au 10.561102158767544879 15.407403459442237548 13.735685108279566791

Au 11.159725244658138976 14.745749883336571884 9.140850047187308647

Au 9.696945255794178209 13.795380420282276290 11.753037750811424544

Au 10.258797501305597422 12.205416068811851815 9.477464994847897728

Au 10.594779258737093031 11.132515669198541630 11.955817837586749874

14

Au14(7)

Au 15.812090123287923404 10.008547527043422321 7.500000000000000000

Au 18.399993392173257689 17.741208186439720862 7.500000000000000000

Au 20.121580049627439735 15.618949959751706302 7.500000000000000000

Au 17.501392245268547754 15.133741625290991095 7.500000000000000000

Au 19.376429462582340335 13.026852765437162418 7.500000000000000000

Au 16.643583896433252534 12.556467548438204673 7.500000000000000000

Au 18.505126772716923966 10.521363446196099645 7.500000000000000000

Au 15.744879075752972852 17.264884784823543384 7.500000000000000000

Au 13.158302511946001090 16.693448569052677044 7.500000000000000000

Au 10.568693092316291171 16.282065184678895520 7.500000000000000000

Au 14.873133836047133016 14.616903538740256607 7.500000000000000000

Au 12.067162062800207423 14.066604780844631506 7.500000000000000000

Au 13.960939382717981516 12.028317961247298129 7.500000000000000000

Au 11.381220120329709644 11.492618358015080915 7.500000000000000000

14

Au14(8)

Au 17.575969325158212087 15.298983695069690469 7.500000000000000000

Au 14.910200628871553619 14.774295827172831963 7.500000000000000000

Au 19.296571581480488788 13.199175393166818537 7.500000000000000000

Au 16.642152371652176868 12.710978203489016636 7.500000000000000000

Au 18.378519473515996907 10.621235074933277787 7.500000000000000000

Au 15.743700474086136154 10.057244729931476002 7.500000000000000000

Au 13.159579058826521347 16.873924657135948024 7.500000000000000000

Au 10.567999223552021704 16.295730247119028178 7.500000000000000000

Au 12.288742788258861793 14.207682006028401034 7.500000000000000000

Au 9.668981007359835900 13.538868027191433896 7.500000000000000000

Au 13.995892449322031581 12.103148802248492544 7.500000000000000000

Au 11.384853939149735425 11.506350953397021186 7.500000000000000000

Au 13.114678810260025799 9.421729356947304623 7.500000000000000000

Au 8.020674277506529393 15.627220198168918941 7.500000000000000000

14

Au14(9)

Au 12.260056811837328539 18.512233425484325267 11.341905370353265070

Au 12.267293573510423599 16.574045262598836814 13.228805836020756104

Au 13.619805679901888951 15.830033378261882504 15.393405170024035300

Au 12.961663312531326753 15.985286674975217380 10.491055941798551387

Au 12.669975142978184834 13.876080922124391037 13.861187617916483461

Au 13.354666829917556825 13.394303108547299885 11.097676644906179888

Au 13.329185151123168040 11.398931464458303964 12.997932289373142822

Au 10.289650618986881270 16.611330188718703482 11.229660614776150851

Au 9.126590448329448435 15.787219512842620261 8.907889542471519206

Au 10.036757818512839435 14.854449310564575626 13.512482233738554172

Au 11.323862662304255977 14.090378561482975073 9.422072278125488864

Au 9.052680802130559812 14.142127624573650024 11.079869241238249344

Au 9.715815761653107785 11.807774365562991647 9.838671544909903588

Au 10.918466920283256272 12.433346273803909554 12.201349875348018514

14

Au14(10)

Au 11.034404059358934092 16.045305928914601168 12.700933826453585596

Au 14.323706133372702709 15.332209671115473171 14.179226810462141728

Au 11.891251258407681490 17.044932551584619063 10.272150096044732948

Au 11.726669858450792461 14.567925551446725407 14.844434705873648994

Au 13.292781155721447917 14.726096217269438071 11.537390452345231751

Au 13.914933225510832671 13.334233803600509916 15.974432260639028769

Au 12.010582153225289659 12.318675950233455296 11.155762226187329489

Au 13.415155594339861267 12.683744677366433962 13.380806397032330324

Au 13.675325172557066367 17.257081919538510562 12.470452056298796606

Au 10.720724544408525958 14.651788747762855891 10.175893886114463882

Au 9.326043470205695129 14.215175851421054887 13.700290827628112567

Au 9.156221896154344009 12.662090776705710482 11.410095227586470656

Au 7.249324403409338480 12.610286361762598872 13.215548234511588888

Au 10.247087861877357540 12.216986374277658101 9.022460819822240907

14

Au14(11)

Au 13.928078798801555749 15.003328542471191298 15.615092837240045043

Au 14.043799115610347883 14.090459098859218656 11.061738355225042341

Au 12.698450035510225220 16.395423407115650605 10.669975370934636771

Au 11.490697720001843862 15.390166577841155160 14.440998798228818600

Au 10.347551638532156204 16.568617924483053372 12.301296249070098554

Au 10.323817941876543003 17.258412242750502230 9.659614356020693293

Au 10.501834133677562022 14.600719320602179252 10.258729519135346919

Au 13.221332389950912756 13.266494348180206231 13.684455218914440167

Au 14.198021502954967232 11.481948804041795853 11.920356090687022288

Au 13.912481677888962395 15.976409859928818236 13.041945319716951701

Au 11.706419662880660937 12.482720133750147440 11.414364363962404170

Au 8.945398107295968515 14.849929756902474409 13.832202672284388711

Au 9.094426743311037953 12.892862258738041703 11.797848266694636266

Au 6.953590062707543318 13.161844845334879750 13.346610039885179688

14

Au14(12)

Au 15.909040152717953021 13.142865302507773961 7.500000000000000000

Au 18.537813187425651762 20.807633972619171914 7.500000000000000000

Au 20.463216172075821220 18.851235473612405258 7.500000000000000000

Au 17.751002195918658799 18.266005000373905176 7.500000000000000000

Au 19.591753167746549735 16.276163544295403796 7.500000000000000000

Au 16.849816168546777106 15.674450821550772517 7.500000000000000000

Au 18.783411476399709983 13.780408248120179948 7.500000000000000000

Au 17.799093143572715547 11.309103589439445159 7.500000000000000000

Au 15.903149983101638298 20.405884720748545647 7.500000000000000000

Au 13.261129693605228752 19.728694600100080692 7.500000000000000000

Au 15.086185204045603925 17.753709959129533758 7.500000000000000000

Au 12.334614496960451646 17.217051461349448971 7.500000000000000000

Au 14.154958995014402134 15.162065145575985170 7.500000000000000000

Au 11.534276434868324657 14.686777509577211731 7.500000000000000000

14

Au14(13)

Au 18.460415753328970112 17.849345919031456731 14.798494000979102836

Au 16.062090291704770095 16.859824668077425969 15.519748610386203680

Au 20.247279408425686853 16.259732626561667956 13.797098790016317338

Au 17.876293061829255038 15.067174463029738618 14.458326785775348355

Au 15.302623873763156581 14.283658992996015158 15.307402295440695639

Au 16.707580757812394268 19.600723799536698522 15.811739182722325481

Au 17.043438683518289167 12.511428632277935336 14.258344231603782504

Au 14.311368823596367861 18.635590691833932908 16.578107361014200904

Au 11.829128519296782329 18.017723153312914519 17.425722533004876169

Au 13.551959076569216833 16.046039937858033397 16.349801491056137337

Au 11.105121830791826909 15.443958719118871414 17.301286707224782901

Au 12.772105912610408396 13.400810691664956309 16.187182313910575715

Au 10.270448667595120540 12.990793341657280280 17.213538737088530439

Au 14.547219586157872584 11.704887683043796898 15.075362306777048360

14

Au14(14)

Au 17.539146156427342760 15.327598242710941179 7.500000000000000000

Au 14.914709164579539546 14.683019506289712197 7.500000000000000000

Au 19.260030714127875484 13.233002401121492397 7.500000000000000000

Au 16.692942505083337323 12.640664991199075828 7.500000000000000000

Au 14.049215950333421077 12.018067621633779396 7.500000000000000000

Au 18.640104990047898781 10.608408076333986969 7.500000000000000000

Au 15.980788773547516257 10.019524259647315745 7.500000000000000000

Au 13.424006546008628860 9.372987703020243089 7.500000000000000000

Au 13.079626652915404605 16.669325404412440150 7.500000000000000000

Au 10.428118782498584594 16.074663298114529653 7.500000000000000000

Au 12.275154632638143326 14.050600607641730022 7.500000000000000000

Au 9.619850096821933150 13.518665387662455402 7.500000000000000000

Au 11.353326355002836934 11.436452886661411910 7.500000000000000000

Au 10.858834647967627873 8.822337566551016863 7.500000000000000000

14

Au14(15)

Au 13.251542701925892231 16.329825395546574640 13.600849179768754738

Au 13.945397953107237399 14.059414626388386793 12.003361723454062826

Au 12.909267375310461645 16.167846619414014242 10.768900273795940947

Au 11.627626157853994115 13.829493987883015649 13.759275282939276153

Au 10.515108942479040266 16.078566043439334265 12.438543241942895179

Au 10.569675308767278565 17.364854740490294205 9.989061014422034646

Au 11.002155773057912924 14.674804438239231530 9.507548468840861133

Au 14.079776085174366074 14.044170335899805124 14.891152879569313328

Au 13.366626804950122676 11.723483949297941464 13.318745271260150176

Au 12.158035003265052154 12.308839437814860673 10.903110799134084985

Au 9.914207434031505173 12.167876426041374316 9.339281661546742441

Au 9.009993566065309878 14.506174024839546988 14.072959790704542726

Au 9.730194307197265857 13.469456696936433104 11.694799671267142926

Au 11.930195918815764244 18.385589964769785354 12.334025588353073388

14

Au14(16)

Au 11.453106650267729449 17.932379739182771061 10.599875765730246258

Au 12.964002822114885305 17.417908954779246500 12.783456095810766584

Au 13.920859508276762995 16.195488929902293052 14.990221255128883371

Au 13.131078102258408435 15.715873521571081639 10.314659483338006751

Au 13.457380280652557758 14.625177741627153338 12.768950461561473730

Au 13.216674623616114559 13.585304961545505265 15.269429123418738925

Au 12.704975963305869868 13.084841388881468305 10.645682877026583668

Au 12.164752284294246820 12.121078307979063382 13.289779471861749016

Au 11.763303389044235558 16.811629339206206168 8.143916474968829533

Au 10.623218220202879536 15.979872453351243067 12.492832829199624456

Au 10.311663357368042071 15.256811362610090299 9.820209909270561255

Au 10.096497223392910669 13.429558537230471771 11.874514826385599520

Au 10.408503610285233520 12.611973582289078166 9.146767106593223673

Au 11.032847930921366952 10.847619149844256015 11.175112024705191160

14

Au14(17)

Au 18.196572919665310764 8.268679066254223997 7.500000000000000000

Au 14.929217370972068579 14.702059805997919284 7.500000000000000000

Au 19.391363661146911568 13.363198808646160387 7.500000000000000000

Au 16.818739691814155179 12.787722177865278539 7.500000000000000000

Au 18.789938922956118006 10.797506760991495867 7.500000000000000000

Au 16.120899457580957659 9.995889015394535448 7.500000000000000000

Au 13.039582925332208418 16.616660455513262207 7.500000000000000000

Au 10.411281725807736365 16.012969274465028491 7.500000000000000000

Au 12.340498860523776870 13.973767329828813644 7.500000000000000000

Au 9.733791824493222222 13.396200034332288453 7.500000000000000000

Au 14.247494211580610468 11.992635493116281964 7.500000000000000000

Au 11.481761609810078539 11.361225211341375640 7.500000000000000000

Au 13.536530331911539093 9.397364408488346754 7.500000000000000000

Au 10.993183320404948944 8.748345024764892841 7.500000000000000000

14

Au14(18)

Au 12.399077557225448132 18.517301056464511078 11.023509273712184253

Au 12.132331938319381948 17.253294763800067102 13.386603133342013905

Au 13.217477189669097015 15.895367763381965887 11.158074031901895395

Au 12.300013014436251169 14.891491529986794262 14.965700116931799357

Au 13.885881221630421933 13.912354449511690646 12.858919693464509137

Au 14.369209106823719324 13.271154481954411608 15.490142509886981159

Au 12.196800804961384301 13.413951919157529602 10.830200134029896120

Au 12.141280504073225899 12.179553718944170271 14.240185843123191134

Au 10.918817645342521416 17.031815452569233571 15.780480382094033587

Au 10.430995489443443347 16.607935242043708968 11.163725502933706579

Au 9.889412375086795493 15.508725406008901260 13.670423857763340791

Au 9.497799400656564472 14.118730189360297445 11.434573614167046429

Au 10.043781489339803414 12.218371077244658096 9.589163814333657498

Au 10.590493698991211957 11.608901830572474267 12.221346395315634936

14

Au14(19)

Au 14.032404584571246176 16.181203366023222401 14.857957722639845599

Au 11.750664000892363958 14.902728958310072827 14.109971589655652124

Au 13.993003633622477011 14.870355370289605190 12.443346538127235235

Au 10.169786844836526285 15.532706576520997288 11.873124817537032527

Au 12.482278439452191066 15.469462884234188138 10.305776960407875720

Au 10.665531186361711491 17.517709739323844786 10.081714717064301112

Au 9.942945204795666569 15.007198077110802004 9.148235225577643348

Au 13.972342717355040520 13.407691138588743840 14.864607752241765226

Au 9.076008256664572116 14.525843372951067423 14.129041863331973872

Au 11.398201833843806696 10.466518969680326023 11.353739844953919302

Au 12.885423568703366470 11.476713837539486818 13.440810001670278950

Au 10.556478837908214174 12.823163188448001293 12.602722084143330505

Au 12.894864800890532663 12.877095070582353031 10.974059323879826522

Au 10.333517060102382246 12.489409370397792642 9.815688788768826001

14

Au14(20)

Au 16.505933340799401776 19.789329075650790912 7.500000000000000000

Au 18.212817994207583183 17.838914080357273662 7.500000000000000000

Au 20.048614680527172993 15.888615168361029717 7.500000000000000000

Au 17.367176692702543050 15.253283099258764466 7.500000000000000000

Au 19.281958928103179574 13.321764197369224902 7.500000000000000000

Au 16.539402376004424866 12.522207675215870637 7.500000000000000000

Au 18.631256432248317623 10.812811312157563748 7.500000000000000000

Au 15.403456543034639736 17.322805414155983073 7.500000000000000000

Au 12.882151007924118602 16.544930238840343861 7.500000000000000000

Au 10.290986578694955256 16.111241331979822888 7.500000000000000000

Au 14.768287258372062354 14.637746177908889322 7.500000000000000000

Au 11.853192612547781337 13.946224548303222335 7.500000000000000000

Au 13.937376019343997058 12.087065439798976740 7.500000000000000000

Au 11.426564210736080085 11.310993558116800628 7.500000000000000000

14

Au14(21)

Au 14.648293265988332479 15.842843601620586469 14.653179162946594616

Au 13.924376928760441530 14.362604163934737045 12.478954315022766863

Au 13.829031513679145959 13.212325184188426164 15.004735048591159696

Au 11.902726907926297173 12.332275937747386507 13.369041415010039842

Au 11.983413500772249094 15.294607615041101667 14.393280626130248834

Au 10.543889376707015870 16.242545165392495932 12.340516651036569584

Au 9.665401601266324860 14.085924329247635711 13.784729201163960610

Au 8.016272610056834225 11.995497331136578012 13.449121755670679690

Au 12.796333571532167994 15.928496373828767929 10.647120237221868067

Au 12.386900923581915634 13.101249359164320651 10.686381320523434368

Au 10.402673759518505037 12.294144304817715252 8.902309467232566220

Au 10.576017162441758046 17.425396687291240028 9.857574151207497692

Au 10.245103292871686307 14.785587746500082673 9.899524875935417967

Au 9.718438080896778075 12.457905761088818863 11.494701857307491721

14

Au14(22)

Au 14.381971245805958759 11.633303652944089635 12.603184907597757558

Au 12.546420601421489849 16.355316031706969682 11.170105146976608168

Au 11.795600056542081546 12.256811752261899784 13.252142243395457655

Au 13.597506501328245676 14.301554592512612629 12.558210630041982014

Au 12.313836507630773198 10.019499080561066862 11.518929957672744635

Au 12.961389898868777237 12.507236533971568093 10.363671081768536908

Au 10.972595389625125151 14.961135046592666242 12.928692111236912865

Au 11.244003137067139164 16.965337221357373210 8.751715150862878545

Au 9.748470604620729674 16.483027799268672453 10.929243853381370499

Au 11.177120255185199582 14.350122165464300750 9.483239354591406212

Au 8.316498380597352025 15.515072999134297049 12.958141792033993411

Au 9.089741706678079680 12.899154554537718198 13.178959332194745357

Au 9.884416641869879783 10.295226281276859126 12.734660108577800841

Au 10.302334241759268707 12.087513132409274164 10.672033683668589177

14

Au14(23)

Au 11.949208991155920501 18.322327683205234194 11.437883264425224183

Au 11.595691087679115938 17.158365064257761645 8.961959800989758307

Au 13.563142790731037124 17.181763173369208886 13.297476904332011571

Au 12.797952271480326658 15.708321683583498896 11.081862821666181418

Au 13.389492132483965747 14.493462282598226665 13.439347365603591555

Au 12.245225844172988872 13.070580656529942942 11.276511216763346113

Au 13.700072710324818104 13.202022692502348278 15.726923814907081578

Au 12.258124227973414833 11.985917275319224018 13.837790339223326797

Au 9.961151940330065457 16.342597666019774039 10.981758698201474544

Au 7.966021818654441589 15.448679728536820477 12.448469314514651884

Au 11.231807098775096421 14.440778469382898663 9.088710385274486825

Au 9.351032123395299323 13.612847338228339922 11.150766519633158680

Au 10.229627025385806149 11.898379668297065948 9.265060481668989212

Au 10.522601284457438808 11.129148473169774292 11.959997228796517277

14

Au14(24)

Au 15.896182340102670949 14.591174823039390063 14.481932186835850374

Au 12.425048149762952576 15.372404662004036879 10.661466682714584309

Au 10.803654739085178704 15.195889284144938003 15.827208575033496984

Au 14.141520762152223867 14.270404665995526372 12.499188530303037226

Au 13.383839835022470766 14.903175912166556571 15.205530916701226474

Au 12.260098784454825704 12.714636500544552078 11.242950604944756066

Au 11.482997871152024416 14.600908863013353667 13.252207367549324601

Au 10.979624125017071634 17.517215589469287806 9.786667862056265932

Au 9.593369586375825264 15.694765252459040639 11.275043540832022870

Au 10.412197182956042241 15.017365088041556120 8.706954204719506762

Au 8.824270798978229635 14.872811564064408074 13.758810838993763781

Au 9.425801115379320194 13.021392911289241923 11.850553016070588086

Au 10.546781494037034577 10.591696163096218086 11.294520586976467769

Au 10.256518384524234477 12.466469564671362136 9.259894440269754057

14

Au14(25)

Au 14.116971097495619958 14.968989218652396289 7.500000000000000000

Au 18.575348837465345042 20.633397757718334731 7.500000000000000000

Au 20.395505345646697037 18.583544278869304378 7.500000000000000000

Au 17.715415267451909642 18.010417068077096303 7.500000000000000000

Au 19.570980577486718488 16.038164085983765972 7.500000000000000000

Au 21.284620115222587344 13.888543934995322360 7.500000000000000000

Au 18.696585582765724354 13.490134788077522643 7.500000000000000000

Au 16.750256465757871638 22.609933674328544839 7.500000000000000000

Au 14.054129908583862019 22.182841519301558009 7.500000000000000000

Au 15.886598011755637572 20.065341633025489898 7.500000000000000000

Au 13.199829902863328357 19.632580557585168179 7.500000000000000000

Au 15.052367022728523338 17.528048606166457546 7.500000000000000000

Au 12.374473226916698820 17.046795660853831578 7.500000000000000000

Au 16.777269169858794129 15.371510177365895089 7.500000000000000000

14

Au14(26)

Au 17.488602449910430892 15.368130161428570801 7.500000000000000000

Au 14.854647117724095295 14.860931345144615889 7.500000000000000000

Au 19.498264585538812810 13.303941594793437631 7.500000000000000000

Au 16.789464227785600769 12.797440489563571830 7.500000000000000000

Au 18.725516342287029659 10.779378260358939201 7.500000000000000000

Au 16.066941289039753826 10.171829405017728476 7.500000000000000000

Au 12.832992976357097348 16.671756830137795191 7.500000000000000000

Au 10.183998489554696221 15.942441324292179416 7.500000000000000000

Au 12.160571016074765538 14.090838497253372452 7.500000000000000000

Au 9.498394490185004813 13.335085731760583982 7.500000000000000000

Au 14.141210199975075668 12.205474925203182224 7.500000000000000000

Au 11.453076183920117614 11.469188385479130332 7.500000000000000000

Au 13.446034885215608412 9.615340126470453441 7.500000000000000000

Au 20.087875298431672633 15.885437739096278875 7.500000000000000000

13

Au13-(1)

Au 17.900422356624268616 18.634543758043783157 12.631853218771333047

Au 15.922181666758930874 17.019497086746259384 11.813801666178608940

Au 18.957434343414803379 18.715294950391225370 15.141108315261440964

Au 12.881225642806464649 13.594363591691417170 12.984129503346681034

Au 11.804226910509317605 11.877280385187017231 14.691759283729112440

Au 13.781996716304178108 13.475205359399081928 15.614204062316044741

Au 13.890096108880962689 15.278960250132790577 11.175610294963764346

Au 16.856024028529780878 15.301506890456369803 18.765090328788012641

Au 17.916725458757632339 16.997912377186882082 16.997099460166012364

Au 14.828829593365504991 13.558639878781297838 18.141502843185339344

Au 15.812428684540609680 15.224283875004934075 16.252717101458930671

Au 14.903832344473865845 15.300978370646831195 13.711207885034855991

Au 16.897586735033872429 16.958427682331286945 14.473253788799667063

13

Au13-(2)

Au 13.734354892054971842 14.061621231841799684 15.909148525847511380

Au 15.948437801287084881 15.499107381938083350 16.417825816194021371

Au 12.469100115953384744 14.147542979068260749 13.428968812196586669

Au 17.164455493283401921 15.355798613962264199 18.905740675239059101

Au 18.237124325490523091 16.979597537765656057 16.919099279442253447

Au 19.396307611847966257 16.802386082161191894 19.345229773094249737

Au 15.814327842310342831 17.193804047716803041 12.098607845773193148

Au 14.761396771181976462 15.619904611431573827 14.008702393666711217

Au 13.547422438417601853 15.733285666281984660 11.539513896001700743

Au 17.084532310675211164 17.141192467649062792 14.516223543260627693

Au 12.712091184636705066 12.493801874920121620 17.839755719783155996

Au 14.960818519492278256 13.919258570665192565 18.449326496572055589

Au 11.473179871368861527 12.583821998597811032 15.380652283928888835

13

Au13-(3)

Au 16.880756337139100509 15.472306699575428368 15.959995446659750229

Au 17.981814637688501080 14.166417346572101721 18.111280826286211720

Au 17.508093460758718862 12.837640868809650385 15.855915816641617866

Au 17.349359305130285236 16.909887424474501216 18.343508580389826790

Au 18.424299369854733044 15.524261974193674263 20.356722054207825323

Au 16.696417346297383943 19.486449624360925981 18.432072750523563087

Au 16.248217805799122004 18.083690634334018910 16.111754089597546624

Au 15.573500428352483382 20.750791991872731046 16.210532341869281936

Au 16.016805261543634487 22.075206092838417504 18.483011348068426827

Au 15.130842948850739660 19.402444706864333313 13.949874804754355040

Au 16.338160804437819706 14.187946661959694339 13.574700992847390069

Au 16.966374288575927665 11.612594281111508820 13.534065496676689122

Au 15.750557705571258893 16.761698693032840879 13.753036751477857536

13

Au13-(4)

Au 14.640465764631747092 15.791482739392026957 14.079007681359270165

Au 15.738648526082645418 17.289695161535224344 12.106332654153755257

Au 15.699645460512227757 16.088530207333828770 16.512897832306247636

Au 16.803394873804865739 17.593262886952246049 14.516234834050273150

Au 13.525293348199786791 15.463523158241986977 11.582157746589201608

Au 14.701972374243577946 17.001271245463978943 9.705143156083881451

Au 12.492981899619518771 14.025440384483067291 13.547786396979660140

Au 14.692740738881168383 14.655069677314475740 18.594895941457352251

Au 16.747267312055971189 16.379473374088021842 18.946685779707994612

Au 13.535149714114748321 13.149218924709744627 20.486488742272786823

Au 11.447690875789827913 12.549652535017719046 15.586927153102317689

Au 12.541272868470377588 12.891923884476895523 18.039079080197090832

Au 13.577856805594009870 14.326878910990629379 16.037679938739742624

13

Au13-(5)

Au 13.717572080312509186 13.101334056873239930 14.818690597088602701

Au 15.530873839669926184 13.496578401899196109 16.731706159388330946

Au 11.227773382947706438 13.699903972686900744 13.819381770819026301

Au 15.877001506451284385 14.752895238334755135 14.286903302651220216

Au 17.446775673705232634 18.426053236189243734 12.045158281099809017

Au 15.289220835827682876 16.886677725131484351 12.416614228998392733

Au 17.231516244564680562 17.309927170524083806 14.468120730716609756

Au 12.247965627693107038 15.382558558733666842 15.738830637432208448

Au 14.442040042195074534 16.980521335262274363 15.187503244158195415

Au 13.248563411804454404 15.331583612027035457 13.004950539230065942

Au 16.197706175381206606 14.781889718341336248 19.068240341128248616

Au 14.060616589153397626 15.777309837183617347 17.653314215997998105

Au 16.816059948294100224 16.108825551813502841 16.773161836291084370

13

Au13-(6)

Au 16.507813166472899979 18.842056607656388678 17.927828191199878916

Au 18.542203509003101658 17.152567160309104111 18.617414853925300378

Au 14.063804350910714902 19.880290840106759731 17.744351633374385813

Au 19.088504964475959014 19.015101031180492441 13.666373262619076812

Au 18.608879375268308820 18.478558297253297127 16.228786666893313395

Au 16.778455408799075599 17.775269574617848178 14.126650236383044401

Au 14.954266972361073940 15.772559153445733671 14.488945758049691648

Au 14.678136008102267596 18.137531775829522473 15.828424706201090544

Au 14.317132956128832078 13.212708810872618059 14.268910202714781832

Au 17.726392952824234328 14.352946430477889095 18.227176252692299130

Au 19.416494314901193263 15.176570412312248237 20.146573160670335767

Au 16.034345315330071458 13.702085863088745654 16.276378077817035717

Au 16.760469208421515219 16.308380901849428568 16.594743114460122513

13

Au13-(7)

Au 15.911050071150045326 17.123553401368063476 12.064993241389094791

Au 18.119512632153497833 18.661735959883809954 12.448840667484720512

Au 14.714076335836088205 15.403943088726364863 14.038043835296315720

Au 13.774244288432241135 15.695984357181625768 11.529002731030317364

Au 20.418383447167013145 18.508299647354593986 17.181651269487684175

Au 19.233283556426993499 18.542851044062484078 14.852152511539186364

Au 18.229362821167448772 16.922082994879062312 16.987674880345533523

Au 16.008552523637757048 15.433775370932714566 16.438015343270066637

Au 17.017242120179211895 17.007046340644023985 14.492455429565755054

Au 13.768757553568532259 13.861899544268670326 15.984562234996568364

Au 14.998574271234703659 13.798221141631422881 18.463994475667849571

Au 17.142567852448557630 15.303140230570761560 18.919911928512714638

Au 12.851715260597936563 12.289397403496490568 17.999257137414378604

13

Au13-(8)

Au 19.239598620346281876 18.483388680611369637 15.068177418920754462

Au 20.439671470548042009 18.194314882365883790 17.496621906302561200

Au 18.035113359960327273 18.750005002588661540 12.652836756142848529

Au 19.526479907921579837 16.443482343738040186 19.337641603241568333

Au 17.318871071211042079 14.900757219300805545 18.818567373325969072

Au 18.248504872746220684 16.661815980930335002 16.943620163059659234

Au 12.708362690303376397 13.908312543533364547 13.303393319685206109

Au 13.879187368842837103 13.612867403873858407 15.766113246224847444

Au 13.701012516232792038 15.671320525972848969 11.514986707195976834

Au 14.888505251153794973 15.400584479820647488 13.958660925953839893

Au 17.060633233506329987 16.947620876367928133 14.509799930982419269

Au 16.071833178840702061 15.125161687165116220 16.393103880840364184

Au 15.872995742386056151 17.217808389731320773 12.073895587123653073

13

Au13-(9)

Au 16.770552751719069562 16.141541545612945185 16.991926031703293631

Au 16.711564990802322939 17.651706449255364362 14.746729263027914314

Au 17.550575462320590958 14.202536722904463673 18.628229988847724741

Au 15.351217619768274147 15.124757355328453556 14.715543394714902803

Au 15.470063445330907825 16.582231164075164287 12.364625257847402295

Au 13.410628694781971504 14.962633662784499577 12.732838516230915005

Au 17.387024422496313747 18.454874799052952028 12.308480238005616414

Au 11.875735574637865000 17.912438837828041471 16.682156155213224480

Au 12.360223390974216073 15.767204646433221171 15.221145203979519422

Au 14.441341443979329284 17.371426520622144096 16.249080488984862569

Au 13.473388091443272430 13.185005740297025767 15.379607587993293194

Au 15.580193808532198929 13.505746007336423276 16.948705832858546927

Au 11.264220980213007550 13.670262664469166936 13.835876394592339977

13

Au13-(10)

Au 11.709646741217769161 16.434619171968201101 17.879831489523020593

Au 14.282138276564079860 15.776611528694937547 17.641064404501047846

Au 12.453064009882913510 15.367248422570188637 15.545160944886161403

Au 15.740613073430274937 13.605812293333508833 16.800545984446852543

Au 15.249201418514950035 11.166914411152466968 15.841500419479380923

Au 16.381000201308381747 14.836342689154799146 19.222299431911324064

Au 13.224924750899894832 15.164208740436730949 12.890941483403576484

Au 15.148408032849051352 15.703207753184502238 14.848519283885130093

Au 13.920733922879300337 13.185271115920844665 14.699577411809896077

Au 15.430826191912640155 16.834705669022078212 12.382055384913106977

Au 19.035707687529818344 17.744004593088927635 16.327760572226843294

Au 16.994613426657135591 16.143302484671128383 16.916587507531072987

Au 17.258590168353990180 17.305347057801718336 14.358811651482701777

13

Au13-(11)

Au 14.840959478878559707 11.042517858877559433 16.268532959384540248

Au 14.175969599948624378 12.991182078492686358 14.544775965171218957

Au 15.986995939802232769 13.455104758544484511 16.545439715469992592

Au 17.032444881440607531 16.032297484892389150 16.668210847897597660

Au 16.907159794925732399 17.893902050378887481 14.720928960677920116

Au 16.440101816111901911 14.643228117865904281 18.983596038473393719

Au 14.304797964038360902 17.146311264418823583 15.334124213052813701

Au 16.060080737655134442 15.124057794324006210 14.217535581484145979

Au 12.516007036061685298 15.115876807658892744 15.584868563294882904

Au 14.299964226384267008 15.574498971999151564 17.550010942119307344

Au 13.608954011860619815 15.155642338214708076 12.890584825888605991

Au 15.053219161245959157 17.424335743404888888 12.677517330391092187

Au 11.608867323646837377 13.403774511927286639 13.648685626694996742

13

Au13-(12)

Au 15.336530404494897795 17.493237165301458447 12.527647744384500683

Au 13.066907141894287037 18.215080671817872826 11.332006854262967721

Au 17.501707498221250603 16.719382892309862854 13.817134202853075919

Au 12.599579621483735892 14.634174829893327185 15.210052173153464139

Au 15.123103506175301192 15.352543033062438838 14.282955621812529756

Au 12.766048654810145280 16.401096096478916309 13.261129007766269794

Au 16.979071269055427962 16.177172943031141727 16.558315404395411718

Au 15.663784527501999122 17.020999957391229174 18.696113777288140056

Au 19.461041225852774517 15.739371396836332906 15.444572225117354947

Au 17.448257611671092349 13.904624509491650741 15.050443642173371828

Au 12.845344191136485179 12.920138231493719871 17.318854503404413236

Au 14.503545385901221110 15.121092070820282061 17.261451884889101649

Au 15.084686961801457628 12.897777702071632433 15.719697258499373049

13

Au13-(13)

Au 15.411224980802156637 17.511155350034343314 12.339892941236213275

Au 13.826376619885332175 17.602538472928245028 10.208375664676999861

Au 17.650220072181717512 18.938282540262502351 12.796797917984402915

Au 14.557928783465237998 15.613971257747211396 14.385815371107005589

Au 16.022024571878830557 15.363311525488445142 16.694974902964784036

Au 16.749725443893424881 17.116320923892960337 14.699864227731486821

Au 13.096064489959562849 15.869175254257600116 12.099836157133889714

Au 11.680239599833434738 12.424281123872185972 15.906993969867626149

Au 12.316386705994368356 14.143655742897621508 13.919234327373111171

Au 13.842277305170206603 13.849854262957299156 16.363478938978161636

Au 18.276331899282741489 16.836728581758691803 17.150999301832090538

Au 18.950401040217403903 18.511135981636101633 15.200570371697674332

Au 17.375822250435128069 15.066498620267198660 18.971261402416686792

13

Au13-(14)

Au 17.042895852539174939 16.190310643398881041 16.721855226213506285

Au 17.403988578223657413 17.249785249272495236 14.278167809157904955

Au 16.400188945680227448 14.998854587213759970 19.079541915646458961

Au 15.138225607601752287 11.153664355621328852 15.945433742786470077

Au 13.783645582521954154 13.162076387079022055 14.810775693769953065

Au 15.658093352116694064 13.644877750160121721 16.771163975461412576

Au 13.329107270145387432 15.232244745578025658 12.834509541070676875

Au 15.475117584676510774 16.741561856860769808 12.249856070470226399

Au 15.190885036533552821 15.708847384457891749 14.773867801662310839

Au 11.282226803404068249 13.677898616220343087 13.721987992411962765

Au 11.745897405771572153 16.718570715076012334 17.815573405528127182

Au 12.404331989761754329 15.421188137678722541 15.570918965762853503

Au 14.278368813023735839 15.904256143383081579 17.532059555058339129

13

Au13-(15)

Au 15.044514743749303420 15.422534181748964954 13.800069634457294399

Au 16.119933913119435687 15.249854623891060612 16.332148599016022672

Au 13.786701406324496588 15.390875950566138997 11.448823083625210728

Au 18.567215298660823208 16.714220265459363901 16.865947924230763277

Au 19.478970309534879846 18.119686621973212226 14.749947844355054016

Au 17.266809464930243934 16.726875337911746300 14.394614160149037829

Au 11.629648349073725910 12.542576548966140493 15.384090338967213896

Au 12.720542391484617184 12.361716397652136479 17.970353805618167087

Au 12.644998680062359497 13.947101503743894568 13.351029133322949605

Au 13.853411870322092980 13.877682153897612949 15.876903625022984912

Au 17.222008017395424417 15.105236534494107303 18.789760413139934059

Au 14.990771780704644556 13.750071562072697873 18.381470513373415088

Au 19.471141983638275974 16.404845189622943025 19.359140455722172902

13

Au13-(16)

Au 17.291785883459741768 13.760490167942496242 18.303846128566149787

Au 15.626756657144394325 12.538582453486103674 16.546738333524928066

Au 19.262139353101360939 15.372737348295270721 19.282409388064003508

Au 15.147149055668052142 15.165332725058945584 17.190433658896257185

Au 18.552265812992725813 17.716199974936252204 18.079661366871064843

Au 15.956937376021681274 17.693552815393978506 17.045053240951254736

Au 18.780745528611753059 15.373651595550914095 16.581193454316274227

Au 14.246798663127044904 13.855674817563720325 14.517852462250276702

Au 14.778456417061839545 16.543665539108452833 14.787822714062135887

Au 16.898339286465688502 14.445848289611294746 14.899022062506171338

Au 15.889074569984655838 18.998275323204342868 14.217078913911272764

Au 17.842018553582736473 19.475096424249461080 16.142247004867670057

Au 17.719804843777925640 17.025477125598520445 14.743823098213111322

13

Au13-(17)

Au 17.050755549571427139 16.927212294982176388 14.496733276580810568

Au 15.854288171970388532 17.198843263104265588 12.111930095402136942

Au 18.305335440013571713 16.689352068787368211 16.862401710054026438

Au 16.016492773917782699 15.301091564837259185 16.469796256012262603

Au 13.615059164392691926 13.919645080417245353 15.868118258971787071

Au 14.757136704254028459 15.565409612313395371 14.013247493993347348

Au 12.592138692751721152 12.351900225316160942 17.732001754882116273

Au 13.490506455089263937 15.797604782751767161 11.605714580008161363

Au 12.427566057910949482 14.199859407091286911 13.487591459440325892

Au 14.451914408987276417 17.336967606871898084 9.707067151173454533

Au 17.128533559691408783 14.925056261098989197 18.912798006635142656

Au 14.877065863135566559 13.646133180422028275 18.340706504461337545

Au 19.376766119313614922 16.280145158006448014 19.293784181385309040

13

Au13-(18)

Au 12.667189882726798800 12.357008018697566598 17.698837557999134162

Au 14.936700061708885201 13.696978032666745406 18.341103359750590585

Au 13.665449162300019381 13.937206913139927877 15.851287073193756072

Au 17.155419385940753330 15.072976106148830766 18.824876321276544644

Au 16.040609048588354568 15.388151861839803658 16.403910422023411542

Au 18.357243464118322862 16.790415238452901292 16.949391925780375345

Au 13.443117523280943004 15.794156329296287211 11.701756750577235522

Au 12.372455579224499900 14.145384191120664141 13.460036502126717650

Au 14.355480681319892966 17.400134849616815558 9.837183996201989800

Au 14.750836205181178329 15.582079917733329566 14.039923831062633752

Au 18.123194316625983902 18.609108222130764432 12.710034917743865179

Au 17.087079770997945616 17.012067322706492689 14.544771739749672079

Au 15.844957483986343405 17.323644441450401388 12.082092577514243814

13

Au13-(19)

Au 18.658463398484439466 16.853651191908635809 18.323053539328782335

Au 18.034931004313719427 18.275765780597613031 16.193549857459938579

Au 17.314111057822834994 15.581835223255543710 16.261094997052413902

Au 18.265012682317312454 13.998567530537622261 18.305269515367761812

Au 17.253194187625247480 12.810851848129129138 16.174441773726400129

Au 19.404554594479947127 15.304151824132672388 20.347464668513747910

Au 15.559679112866326989 18.991368027347736103 15.027807625806355318

Au 13.168635464932824775 18.262989520421527345 15.872896077954264271

Au 17.826366396885898524 19.553058085694733137 13.724615956139304629

Au 14.759639515411757671 16.301521405751518756 15.006221271529009087

Au 16.281021439377507676 14.325087627814275137 13.940112698320950457

Au 16.380302464839889609 11.683979749245303026 13.880116588545575951

Au 17.114509856642101226 16.917215745163808549 13.724884273255574030

13

Au13-(20)

Au 15.351315409623749630 11.348937334797319920 15.925662107968706849

Au 13.757102490495990210 13.124939695524652450 14.755944914616428321

Au 15.672580862718710648 13.857440766234896401 16.736183014240218370

Au 15.605612212662865801 16.714850680139317518 12.348512051773820275

Au 15.167044148874463616 15.764928512117871051 14.754166625179244932

Au 17.431123671044939982 17.398628926048882448 14.189253530789418534

Au 13.360529456061069453 15.142141132077762578 12.741101888307214907

Au 12.265823560420582083 15.401563187432419255 15.386573296596786520

Au 11.213560670862282365 13.412017509505712809 13.888961582186684751

Au 11.685330865773130071 16.930116841406245953 17.481915374329659585

Au 16.897090342519447859 16.319819550516225348 16.831819156792970915

Au 14.204164603195982508 16.098241213856365306 17.357404613442298569

Au 19.006422999746771296 17.821331294341735685 16.408957403776017969

13

Au13-(21)

Au 15.619755849897149602 18.348191353279339921 16.281358731183964039

Au 17.066066698031644222 20.054966589034354030 14.627619073807069938

Au 14.734164345934322782 16.364645440679606025 14.679567341082828236

Au 18.096675143868878877 15.798364628932539233 16.043396466905036846

Au 18.989877186248776297 16.886829123605014757 18.435457828859810547

Au 18.405382033862426994 18.536602135134245373 16.456394318681731193

Au 17.508215622306984471 14.425298983385713925 18.537471501476730396

Au 19.103008222281726347 15.226653150946763304 20.521143762892116058

Au 16.551918552741838653 13.397654685476739544 16.251691404085054415

Au 15.491779075088320639 15.801508305874166993 17.254322374891415848

Au 16.822703255199346728 14.792507419015107573 13.725213056796949473

Au 17.284611749765989686 17.437065833253917901 13.907812697583569417

Au 16.092207856772262176 12.221573847382925848 13.899491573754065854

13

Au13-(22)

Au 17.771721047740829391 12.991352741900739076 14.558015905997310213

Au 15.057454616291881422 12.950676358975277580 14.535937333232219260

Au 16.365467522874908468 14.518297756944948063 16.340275187573411131

Au 16.891197981817530405 16.827369546559410196 14.791523336441393610

Au 17.560270384808962518 16.712465695191536241 17.480379500395223147

Au 16.686499612406283433 17.385221757654775843 19.911100821170666819

Au 19.025126620945556510 15.299082258232521880 15.572209441042659250

Au 12.493347835604943441 15.137907731376339271 18.894808803449404166

Au 13.556658795529447303 14.579143183489614799 16.511490534993036761

Au 15.030578714784862626 15.911403881658319648 18.396996114117957433

Au 14.466821361167820470 15.663768763345878199 14.130181218460046821

Au 15.479775756097780004 17.658101298119103006 12.694857169215971737

Au 12.454620475929457868 13.753051683551369422 14.144329394910757358

13

Au13-(23)

Au 12.225062813371991410 15.160977440552418471 15.736334871155547077

Au 12.269278560722380433 17.698343417681858369 16.829045259785104349

Au 11.048573226977783435 12.936319401352086800 14.807013316349522114

Au 15.641482788798086290 17.272274924197546397 12.634668644008460348

Au 17.014976452374579452 17.194309063344409338 15.033546768044272923

Au 14.182225279552236330 16.959943324515872831 14.954344006727273708

Au 16.290873331969905280 12.192902225567729957 14.335395952082262028

Au 13.726878882230074908 13.113347204083593667 14.810748360528013023

Au 15.702875094097342057 14.886664487144363633 14.015670448102641288

Au 15.861402308403713235 14.454529940967953294 19.219413998377152808

Au 17.088904016275535014 16.164728932346928758 17.427207151625101034

Au 15.779163370598892513 13.800133749535646643 16.541583043126603769

Au 14.259400933626888985 15.873344191710362594 17.479665235088216235

13

Au13-(24)

Au 16.698129655597153231 17.286719982934585005 14.479482319162865878

Au 19.162628821247285060 16.886493078191584516 15.463853873334636901

Au 15.311706887362976914 14.873549137482410032 14.831124885542298131

Au 15.309560340285763402 19.260726975300091368 13.282851752619276198

Au 11.948219188037549898 17.433474846813020065 16.680529041120813361

Au 14.007517744913535296 17.493771241881475476 14.824797867390829609

Au 14.291608332636510426 16.113700590222968856 17.259113778769791026

Au 15.999780718735433283 15.335760311688144242 19.255141769677305064

Au 15.447470261916166123 13.443493882712044041 17.334058714583235883

Au 17.068224388294325422 15.898567399173199277 16.827054686017930862

Au 11.478025270273361613 13.011103182993862148 14.078656989967168300

Au 13.752740198342856104 12.647586203592693366 15.496397148777345976

Au 12.489502208357349033 15.171031017014104947 15.244231400037065782

13

Au13-(25)

Au 14.572847947511558431 17.294371020340598477 15.968701025790362991

Au 15.623242222806190682 19.808734369919619667 15.750814916173958125

Au 15.364584945371685620 15.114029218945169930 14.447810916965273975

Au 16.234414182056021048 18.304607238614892140 17.949837951009193660

Au 19.556530680797006738 15.439582788828897009 20.277286769629057517

Au 18.704790834503658203 17.140928713175370035 18.430854626913045990

Au 18.109454543370684831 14.283718826510513722 18.331876082984685183

Au 16.528159255188128895 13.320697245951077292 16.437223004952510763

Au 14.776155660659778590 12.531560639261087431 14.560440415327212449

Au 17.269388640674637969 15.962732183322454915 16.326628158810759572

Au 18.352742603499713425 18.569117962129528365 16.143975330328473206

Au 17.656817880631326290 20.012144461267428852 13.939114468063694474

Au 16.596627922929219068 17.504559870733139348 14.051378547051921686

13

Au13-(26)

Au 18.459765290075253574 16.970301574137323541 18.256504505406319083

Au 19.111177899321351248 15.508184878595796974 20.394352807405244477

Au 16.060470452433097677 17.972259481463119357 17.162218251658842405

Au 16.351350692160536937 14.456351109989405757 13.933075477132323527

Au 17.388252124835421597 16.956683821640215371 13.598406444557651795

Au 16.028618360670389364 11.806759589515358755 14.022643562181189125

Au 17.986939566192123863 14.126111302630215860 18.404635704588613976

Au 16.959374429536783424 12.889620553635873890 16.280132408237058428

Au 18.222181285970176390 15.469572609359540039 15.838373009212181941

Au 15.728786662220610282 15.235303618206026854 17.180730835427858949

Au 16.220444192009153994 19.277757181053996050 14.599323632666694195

Au 14.991070520028118906 16.700990381847788058 14.968277026899459159

Au 18.489379343547255985 18.268746425926039478 15.858061960626681497

13

Au13-(27)

Au 18.982764685983141817 17.729688104535700433 16.410262817188588258

Au 17.389241344784505117 17.406835824217804287 14.312811619832944032

Au 16.888991571657161472 16.181740878682973062 16.907890998442066888

Au 15.414464416366783439 16.774745195605486714 12.342353700130530569

Au 17.545380544384162391 18.372209939596249484 11.860287499832450564

Au 13.344345491983071739 15.158142014183296098 12.992798700770679332

Au 15.140783487034392252 15.729058524191076529 14.851419599778942882

Au 15.362085802878445051 11.234246174711472577 15.814973004760952691

Au 13.627454915838050908 13.049108061051500229 14.824812840438566397

Au 15.653983663496287448 13.713812081883194338 16.715343061242080580

Au 14.193335982850477706 15.903288715650990781 17.470209894235278369

Au 11.683120980211430862 16.734006298193833118 17.702734597826808027

Au 12.203790708532251230 15.178804612496600512 15.559745976519687716

13

Au13-(28)

Au 17.154698315041105872 17.412693044603621217 14.481893946303534548

Au 16.660705638077743629 16.044958394603678897 16.699968114095955940

Au 17.214441109975858524 18.556177846411145538 12.071302069124376644

Au 12.432196874708932199 17.524321594993963913 17.106203743904785597

Au 12.157387525475259693 15.086729405644604185 15.847686497953093365

Au 14.169285484902601979 15.518418527175485977 17.855586292844968455

Au 15.105785796960272904 16.962615020909225905 12.436180986724137298

Au 13.215285037796530077 15.298577882208991952 13.268423340372429209

Au 14.381212213334045202 17.004726500030120206 15.255215615181850097

Au 16.110886433425456232 12.106826821625997326 14.569756015006463556

Au 13.537652203370154069 13.044611422334897455 14.891600804836269134

Au 15.554456226341137892 13.483241109927806889 16.892988595896376580

Au 15.870235643590817176 14.812761413530491694 14.233108497755305066

13

Au13-(29)

Au 18.353888739345155301 16.827736942572872891 16.762350866867514299

Au 19.333659514645994193 18.457624725878886807 14.836345335364113041

Au 16.142711202167493667 15.357106668644581404 16.516222026636715015

Au 12.847405850469780120 12.352003789564317771 17.791430794807535420

Au 15.086379000121580063 13.694984656389472377 18.367199725415456868

Au 13.840945393306775202 13.982221676133599786 15.901617868051440396

Au 11.502092895939615147 12.504072500695688319 15.423890222307017339

Au 15.834363411415161238 17.277140920019380843 12.059718646299279143

Au 17.084954637997086024 17.052321805331910554 14.399630218175811791

Au 14.848934909387637049 15.629493374831742614 13.995727148042631072

Au 13.438431819980412030 15.738374070918260017 11.639232058238176748

Au 14.413377610254936911 17.350178681404766934 9.775372835817632833

Au 12.510820303967799205 14.159184697614335846 13.525139359976623865

13

Au13-(30)

Au 13.778784460318492577 13.217987774133556300 14.860846626092833134

Au 15.557211947086916126 11.314086741420890547 15.565397805359065586

Au 15.165446125331618177 15.755893515519117543 14.871429656122817420

Au 12.405946569159773674 15.373321662751010308 15.655550887403805405

Au 16.995135988835563978 16.207207250338225180 16.826929428560866597

Au 17.332307509115025823 17.319357887945688645 14.418936982309517703

Au 15.671835253589826209 13.613398093162873081 16.880508160500536263

Au 14.242887881967062569 15.859830855037259312 17.705768314194390456

Au 16.327553795906585066 14.913796832119219360 19.180282258311102339

Au 11.887118579042271094 17.076796672416989509 17.683334031558491262

Au 15.402277520510944342 16.877774171512193391 12.283055874312003297

Au 13.352408650998507156 15.311117657875602305 12.962701339701242276

Au 17.616934856137795862 18.401157190767708727 12.017903429573268070

13

Au13-(31)

Au 19.304861050740306894 18.349838619407648821 15.000905954266668729

Au 17.058600942565121272 17.006178508498045687 14.448747082438728739

Au 18.477838256272672623 16.745505075443247733 16.906108308183640077

Au 17.394256113090357019 14.995734998116191150 18.762201385178322255

Au 15.139835373015607445 13.650533340057300791 18.233967268972591569

Au 19.646828347417713445 16.338321742213892662 19.279804902312950077

Au 13.238626229619582020 15.723431492101958895 11.623588998437325515

Au 14.714330201348429839 15.505562683798702395 14.058323333110704922

Au 14.372583305344321047 17.480429763691393674 9.877552387471848050

Au 15.615306104981520718 17.162526710245224137 12.175248963126332313

Au 13.656410316715568598 13.751269708786580281 15.954134317278235500

Au 16.055860373189123180 15.283071742404866100 16.345680513397670808

Au 12.431856304699572036 14.114243012234814145 13.558538371825243019

13

Au13-(32)

Au 15.298500431525829057 16.886144849963859116 12.527857120877262886

Au 17.502427944515495994 18.320762553038715481 12.010101354726762324

Au 13.013071352190330288 15.467504780035179479 13.002328841522253100

Au 15.635834560937727389 11.601592014102353190 15.253897271255587142

Au 15.853203353613983140 13.663594260972741878 16.905318817306429935

Au 14.060981057744836420 13.674394540750656191 14.783585360165647060

Au 14.008132678711701757 15.592771726811120558 17.646472285318097306

Au 12.074049072116608272 15.314331685250690995 15.728160490428903628

Au 16.224807182160787988 14.947146218839385767 19.268455085349593503

Au 14.605390558765071418 16.452036199856351573 15.114625796182737716

Au 18.916762856983485364 17.809802960259098370 16.517789904603748852

Au 17.122544210971227585 17.532112406682838213 14.541785815204587351

Au 16.696132064763521186 16.385774271436496008 16.992957787058649899

13

Au13-(33)

Au 12.098719283538351021 14.090738903132258741 16.559638043316255107

Au 13.442257110719452839 14.788293101559903064 18.724809257445109267

Au 13.479958611734543794 15.082302573519253741 12.695087786287976073

Au 13.745009122664660239 13.057417794903242836 14.601712245166364568

Au 16.265092089607232850 12.248590343912942657 15.044332149115259512

Au 11.301255499969764529 13.938747452847097819 13.930956945312702899

Au 15.816410430855524893 14.952601849103652754 14.274501299233911311

Au 16.141478836906244965 15.334385781600683885 19.028313054694169892

Au 16.948638808876619777 16.100615314820554858 16.558088872919569212

Au 15.148495374387040968 13.939096575351642926 16.858531614968249812

Au 17.498714972890205388 18.434874560758355955 11.809435345929324868

Au 15.537936662791459241 16.638854898067634736 12.114866461865211988

Au 17.367233117058795244 17.220821915422519055 14.194416564745608866

13

Au13-(34)

Au 13.897125019618743025 17.447225330685029121 15.161412352290282612

Au 14.887290860239302148 17.866202639409188180 12.751307111230493874

Au 12.539619836113994111 14.996590136845551200 15.417080306502185749

Au 12.669466358293206554 16.269690196016703965 12.793884080204966480

Au 14.570315767995440481 15.672296580936816213 17.206741194233106995

Au 15.932684939136068536 13.366331798462580238 16.962481724263906102

Au 12.143195889336244164 17.015896340426269973 17.183772472228987738

Au 13.715707994375261336 12.514654053534762568 15.362299699687348209

Au 15.311447048661039005 10.809246176440824527 16.670322834500638010

Au 12.503680153396393493 13.572086161510089397 13.195857311810302193

Au 19.000353854223352101 17.506670507802908787 16.003019276382161706

Au 17.216835309056449432 15.689718732283626679 16.729915159768601995

Au 16.607933681555003602 17.742148714645935570 14.825617985896496265

13

Au13-(35)

Au 12.418256160921773201 17.217989313321908185 17.114890719198150748

Au 12.622507240878071855 14.999399435345276643 15.505307384521691105

Au 14.436316452190794735 15.379475716016843378 17.439358061117253840

Au 14.360508217158812982 17.373661996199402324 15.258107451898926143

Au 13.019599141907209017 16.291713868737975446 13.000323586075825943

Au 14.896155587085372574 18.177922873196365572 12.706094076566010642

Au 12.193640549237533932 13.726834999543680027 13.162788886387824405

Au 16.663116304913444310 17.067506442980565851 16.892906859388347129

Au 16.743010754797520434 18.570095164261928744 14.679375827483406525

Au 16.938364394271758329 14.741223244245150070 18.233214014188074970

Au 14.895521015974397372 10.403027059257498266 16.152456098727292044

Au 15.219708429176863618 12.823465616265773193 17.265234162454465405

Au 13.367071647486584496 12.434808298627533674 15.291227325993174802

13

Au13-(36)

Au 12.423361182568502414 15.303985278344955034 15.581585142570279601

Au 11.845310253834112402 16.916712238967786419 17.652093859438579670

Au 11.458888876085119080 13.556892484671495325 13.795743957920636902

Au 15.416935180805053207 11.140279504176954006 15.993122636860544361

Au 15.729570046468179356 13.661679138816708701 16.791344170523206714

Au 13.827899444183671562 13.026330286051509333 14.927122030341202219

Au 16.209422872115624159 14.931205169538500854 19.222696891735942870

Au 17.036106686457152648 16.113417886208832641 16.903928382925528240

Au 14.316504946489628125 15.946420169158031399 17.449290248451983842

Au 17.373171265109434103 17.061979418522216889 14.343571618132115120

Au 19.132313826145093572 17.625534787466843767 16.240327677306051868

Au 15.830447165391776210 16.682020889524672214 12.262806884837349486

Au 15.083325964347078951 15.548580073551462988 14.640100859956584500
